# Spontaneous Threshold Lowering Neuron using Second‐Order Diffusive Memristor for Self‐Adaptive Spatial Attention

**DOI:** 10.1002/advs.202301323

**Published:** 2023-05-24

**Authors:** Yang Jiang, Dingchen Wang, Ning Lin, Shuhui Shi, Yi Zhang, Shaocong Wang, Xi Chen, Hegan Chen, Yinan Lin, Kam Chi Loong, Jia Chen, Yida Li, Renrui Fang, Dashan Shang, Qing Wang, Hongyu Yu, Zhongrui Wang

**Affiliations:** ^1^ Department of Electrical and Electronic Engineering The University of Hong Kong Pokfulam Road Hong Kong China; ^2^ ACCESS – AI Chip Center for Emerging Smart Systems InnoHK Centers Hong Kong Science Park Hong Kong China; ^3^ School of Microelectronics Southern University of Science and Technology Shenzhen 518055 China; ^4^ Institute of Microelectronics Chinese Academy of Sciences Beijing 100029 China

**Keywords:** second‐order memristor, spontaneous threshold lowering, spiking neural network, self‐adaptive spatial attention, multiobject detection

## Abstract

Intrinsic plasticity of neurons, such as spontaneous threshold lowering (STL) to modulate neuronal excitability, is key to spatial attention of biological neural systems. In‐memory computing with emerging memristors is expected to solve the memory bottleneck of the von Neumann architecture commonly used in conventional digital computers and is deemed a promising solution to this bioinspired computing paradigm. Nonetheless, conventional memristors are incapable of implementing the STL plasticity of neurons due to their first‐order dynamics. Here, a second‐order memristor is experimentally demonstrated using yttria‐stabilized zirconia with Ag doping (YSZ:Ag) that exhibits STL functionality. The physical origin of the second‐order dynamics, i.e., the size evolution of Ag nanoclusters, is uncovered through transmission electron microscopy (TEM), which is leveraged to model the STL neuron. STL‐based spatial attention in a spiking convolutional neural network (SCNN) is demonstrated, improving the accuracy of a multiobject detection task from 70% (20%) to 90% (80%) for the object within (outside) the area receiving attention. This second‐order memristor with intrinsic STL dynamics paves the way for future machine intelligence, enabling high‐efficiency, compact footprint, and hardware‐encoded plasticity.

## Graphical abstract

Spontaneous threshold lowering (STL) originates from the intrinsic neuronal plasticity observed in biological neurons, which plays an important role in a number of learning protocols like spatial attention. Here for the first time artificial STL neurons using second‐order yttria‐stabilized zirconia with Ag doping (YSZ:Ag) memristors at a small hardware overhead are realized, which mimic neural intrinsic plasticity and boost the performance of spiking neural networks.

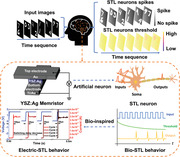

## 1 Introduction

**Figure 1:**
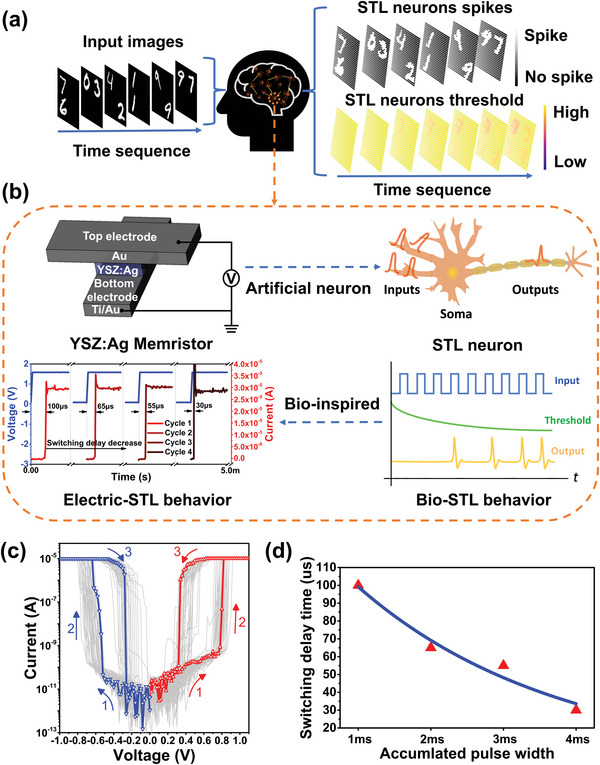
Spontaneous threshold lowering neuron using YSZ:Ag‐based second‐order diffusive memristor for self‐adaptive spatial attention. a) In vision system, the frequently firing STL neurons of the receptive field define the area of interest, where the threshold of the neurons in the area of interest decreases more and forms spatial attention in a self‐adaptive way. b) Schematic of the metal‐insulator‐metal (MIM) structure of a Au/YSZ:Ag/Au/Ti memristor with second‐order switching dynamics, which is employed to model STL neurons. The evolution of temporal dynamics of the YSZ:Ag memristor shows a gradually decreasing switching delay (electric STL behavior) that reduces from ≈100 µs down to ≈30 µs over 30 DC sweeps cycles (i.e., 10 DC cycles per pulse test). c) Consecutive 50 DC voltage sweeps cycles with positive bias from 0 to 1.1 V followed by 50 DC sweeps cycles with negative bias from 0 to −1 V, showing repeatable bidirectional threshold switching behavior with an ON/OFF ratio over 10^6^. d) Fitting curve of the switching delay as a function of the accumulated pulse width extracted from (b). The switching delay reduced with the accumulated pulse width, indicating spontaneous threshold lowering (STL) and the existence of the second state variable.

The brain features different plasticity of both synapses and neurons. Although the former is commonly regarded as the dominant form of neuroplasticity relevant to learning and memory, nonsynaptic, for example, intrinsic plasticity through modification of neuronal excitability also plays an important role. One of such mechanisms is the spontaneous threshold lowering (STL), where the threshold potential at which an action potential is triggered can be lowered by the regulation of voltage‐gated channels on the initial segment of axons, thus it is easier for the neuron to fire, influencing all incoming synaptic inputs. The intrinsic STL plasticity plays an important role in a number of learning protocols like spatial attention, fear conditioning and odor conditioning.^[^ ^1^, ^2^ ^]^ For example, a hyperpolarized shift of voltage‐gated sodium (Nav) channel activation lowers the spiking threshold and increases intrinsic excitability of hippocampal CA1 pyramidal neurons to speed up learning.^[^ ^3^ ^]^ Similarly, due to the slow activation kinetics of voltage‐gated potassium channel Kv7.2, the downregulation of another voltage‐gated potassium channel Kv1 reduces the spiking threshold and effectively raises attention to the auditory neurons losing auditory inputs.^[^ ^4^ ^]^ Another representative example is the formation of spatial attention in vision system as illustrated in **Figure** **1**a. The frequently‐firing STL neurons of the receptive field define the area of interest, where the threshold of the neurons in the area of interest decreases more and forms spatial attention in a self‐adaptive way. The widely evidenced excitability of STL‐regulated neurons can greatly benefit the adaptation of biological neural systems to complex environments.

Hardware neuromorphic computing may leverage such neuron plasticity of the human brain for artificial intelligent systems in the era of big data and Internet of Things. In‐memory computing is expected to solve the memory bottleneck of the von Neumann architecture commonly used in conventional digital computers. The Moore's law^[^ ^5^ ^]^ and Dennard scaling^[^ ^6^ ^]^ that fueled the past development of complementary metal oxide semiconductor (CMOS) for decades cannot sustain their pace as the transistor size is close to its physical limit, rendering technology node shrinking less effective.^[^ ^7^, ^8^ ^]^ This makes the complexity of CMOS computers difficult to parallel that of the brain, where the latter consists of 10^12^ neurons and 10^15^ associated synapses.^[^ ^9^ ^]^ Meanwhile, traditional digital computers are based on the von Neumann architecture with physically separated processing and memory units. The frequent and massive data shuttling between these units incurs large time and energy overheads. In contrast, the neurons and synapses of the brain collocate information storage and processing, which makes the human brain consume only 20 W.^[^ ^10^ ^]^ Thus, a brand new computing hardware is in demand to implement STL neurons to unleash the power of the brain‐inspired computing paradigm.

Emerging memristors^[^ ^11^, ^12^, ^13^, ^14^, ^15^, ^16^, ^17^, ^18^, ^19^, ^20^, ^21^, ^22^, ^23^, ^24^, ^25^, ^26^ ^]^ are two terminal circuit elements that could change their resistance in response to electrical stimulation, regarded as one of the most promising contenders for hardware neuromorphic computing.^[^ ^27^, ^28^, ^29^, ^30^, ^31^, ^32^, ^33^, ^34^, ^35^, ^36^, ^37^, ^38^, ^39^, ^40^ ^]^ The memristors are not only scalable and 3D stackable thanks to their simple device structure, but also process information right at where it is stored through “compute‐in‐physics” that can emulate synaptic plasticity and neural integrate‐and‐fire in an energy efficient manner.^[^ ^41^, ^42^ ^]^ Such memristor‐based synapses and neurons can work together, like how the synapses and neurons interact in the brain, to physically implement spiking neural networks.^[^ ^43^, ^44^, ^45^, ^46^, ^47^ ^]^ However, how to endow the memristor neurons with plasticity remains challenging for brain‐inspired learning, since majority memristors are first‐order dynamic systems governed by a single state variable.^[^ ^48^, ^49^ ^]^

A second‐order memristor may naturally fulfil the requirement.^[^ ^50^, ^51^, ^52^ ^]^ Unlike the first‐order memristor, the second order dynamic system possess two distinct and interdependent state variables, governed by two first‐order (or an equivalent second‐order) differential equations to describe their respective dynamics,^[^ ^53^, ^54^, ^55^ ^]^ which can be mathematically written as

(1) $$I\left(t\right)=G\left(w,s,V,t\right)V\left(t\right)$$

(2) $$\frac{dx}{dt}=f\left(w,s,V,t\right)$$

where *w* and *s* are the two state variables that are physically encoded to the memristor (such as filament length and temperature). The interplay between the two dynamic state variables *w* and *s* equip the memristor with concurrent long‐term and short‐term dynamic behaviors, offering the capability to model complex dynamic behaviors of biological neurons such as periodic action potential, spiking number adaption as well as the STL.^[^ ^52^, ^56^ ^]^

In this work, we experimentally demonstrated a second‐order volatile memristor using yttria‐stabilized zirconia with Ag doping (YSZ:Ag) for hardware implementing STL neurons (Figure 1b) at a small hardware overhead, which mimicked neural intrinsic plasticity and boosted the performance of spiking neural networks (see Table S3 in the Supporting Information). The physical origin of the STL dynamics, the size evolution of Ag nanoclusters, was investigated using transmission electron microscopy (TEM).^[^ ^57^, ^58^, ^59^ ^]^ Biomimicking self‐adaptive spatial attention mechanism in a spiking convolutional neural network (SCNN) was proposed using this YSZ:Ag second‐order memristor‐based STL neuron, which improved the accuracy of multiobject detection of handwritten digits from 70% to 90% for the object within the area of interest (first spike) and 20% to 80% for the object outside the area of interest (second spike).^[^ ^60^ ^]^ In addition to the advantages in speed and energy efficiency, such second‐order memristor STL neurons may pave the way for future machine intelligence based on the emerging neuromorphic device and algorithm.

## 2 Results

### 2.1 Experimental Demonstration of the Second‐Order Volatile Memristor

The metal–insulator–metal (MIM) structure, consisting of Ti/Au/YSZ:Ag/Au crossbars illustrated in Figure 1b, was fabricated. The YSZ:Ag functional layer was grown on the substrate using co‐sputtering deposition, with a Ti/Au dual layer bottom electrode (BE). (see the Experimental Section). As shown in Figure 1c, the as‐deposited Ti/Au/YSZ:Ag/Au MIM crossbar junction was in the high resistance state (HRS) or the insulating state, with a pico‐ampere leakage current. The device manifests forming‐free threshold resistive switching owing to the Ag nanoparticles randomly dispersed within the functional layer. These particles form a conducting path once the applied voltage exceeds a threshold. This contrasts with the conventional nonvolatile memristors with Ag top electrodes.^[^ ^26^, ^61^, ^62^, ^63^, ^64^ ^]^ For example, an applied voltage above an apparent threshold (≈0.8 V) increased the current of our memristor abruptly to the 10 µA compliance set by an external semiconductor parameter analyzer, switching the device to a low resistance state (LRS). The memristor spontaneously relaxed back to the HRS once the applied voltage dropped to zero, illustrating the volatile nature of the device. The observed bidirectional volatile switching was symmetric and repeatable under opposite bias polarities. Such a non‐polar switching roots on the symmetric device structure. The memristor showed no apparent deterioration after consecutive 50 DC voltage sweeps with positive bias from 0 to 1.1 V, followed by another 50 DC sweeps with negative bias from 0 to −1 V. A representative cycle was highlighted in blue and red under negative and positive bias, respectively. The negative (positive) SET voltages spanned from −0.5 (0.7 V) to −0.8 V (0.9 V), due to the inevitable stochasticity associated with filament formation. The device was able to conduct a large current of 10 µA while still retaining volatility. In addition, a maximum ON/OFF ratio of 10^6^ was demonstrated with a small OFF‐state current ≈10^−11^ A around 0 V.

Ag‐based diffusive memristors of the first‐order dynamics have been used to model the leaky integrate‐and‐fire neurons since they accumulate incoming voltage spikes and produce output current spikes when the accumulated stimulation exceeds a threshold. In addition, the YSZ:Ag memristor neuron has an additional dimension in its state space (second‐order memristor), the modulation of the neuron threshold, which is capable of generating complex temporal dynamics such as spiking number adaptation and STL as shown in Figure 1b. The dynamical behavior of YSZ:Ag memristor was studied by applying ≈1.5 V/1 ms square voltage pulses and measuring corresponding current responses. The device, in series with a resistor to prevent the memristor from hard breakdown, first underwent a threshold switching to the LRS around 100 µs after the onset of the pulse. Such an incubation delay originates from the growth and clustering of Ag nanoparticles that bridge the two electrodes. The channel formation was reflected by the abrupt surge of the current by several orders of magnitude, which saturated as the channel reached a steady state. Upon the end of the voltage pulse, the memristor relaxed back to its HRS over a characteristic time around 500 µs, revealing the spontaneous rupture of the conductive filaments. In addition, the observed switching delay and relaxation were dependent on the history of electrical stimulation. For example, with increasing voltage stimulus, the switching delay time gradually reduced to ≈30 µs while the relaxation time rose to ≈1.3 ms after 30 DC sweeps (i.e., 10 DC sweeps was applied after each pulse test). This delay can be regarded as a reduction of spike firing threshold, a manifestation of the second‐order dynamics. As such, the gradual reduction of incubation time of the YSZ:Ag memristor upon each incoming voltage spike mimicked the STL of a leaky integrate‐and‐fire neuron as shown in Figure 1b. The fitting curve of switching time delay with accumulated pulse width extracted from Figure 1b is shown in Figure 1d. The switching delay reduces with the accumulated pulse width, an indication of the lowering of threshold voltage, which implies the existence of the second state variable in addition to the one governs the conductance (such as the size of conduction channel), a unique feature of second‐order memristors. Such history dependent dynamic responses of the YSZ:Ag memristor also share resemblance with that of biological neurons that show complex STL behaviors.

X‐ray photoelectron spectroscopy (XPS) was carried out to analyze the chemical composition of this YSZ:Ag‐based second order memristor. The binding energies of O 1s peaks indicate that Zr and Y of the switching layer have a valence state of +4 and +3, respectively, which corresponds to oxide compositions ZrO_2_ and Y_2_O_3_. The quantitative analysis of the O 1s spectrum suggests a ZrO_2_/Y_2_O_3_ ratio of ≈2 (Figure S1a, Supporting Information).^[^ ^65^ ^]^ The chemical state of Ag embedded in YSZ layer is revealed by Figure S1b (Supporting Information), where the Ag 3d spectrum was deconvoluted to a single doublet with binding energies of 368.1 eV for Ag 3d_5/2_ and 374.2 eV for Ag 3d_3/2_.^[^ ^66^ ^]^ The position of Ag 3d peaks suggested that the Ag was metallic, which was corroborated by high‐resolution transmission electron microscopy (HRTEM) micrographs of embedded Ag nanocrystals in the following section. The large sliver concentration in YSZ:Ag facilitates the forming‐free volatile threshold switching. Moreover, the characterizations revealed ≈16 at.% Y dopant concentration in yttria‐stabilized‐zirconia (ZrO_2_:Y), or equivalently Y_0.16_ZrO*_x_*. Figure S1c,d (Supporting Information) are the corresponding spectrums of Zr 3d and Y 3d, respectively. It is observed that the Zr 3d_3/2_ and Zr 3d_5/2_ peaks were located at 184.3 and 181.8 eV, respectively, with a spin–orbit splitting of 2.5 eV, while the Y 3d_3/2_ and Y 3d_5/2_ peaks were of binding energies 158.9 and 156.8 eV, respectively, with a spin–orbit splitting of 2.1 eV, consistent with literature reports.^[^ ^67^ ^]^

### 2.2 Physical Microscopic Origin of Inherent Threshold Lowering Dynamic

**Figure 2:**
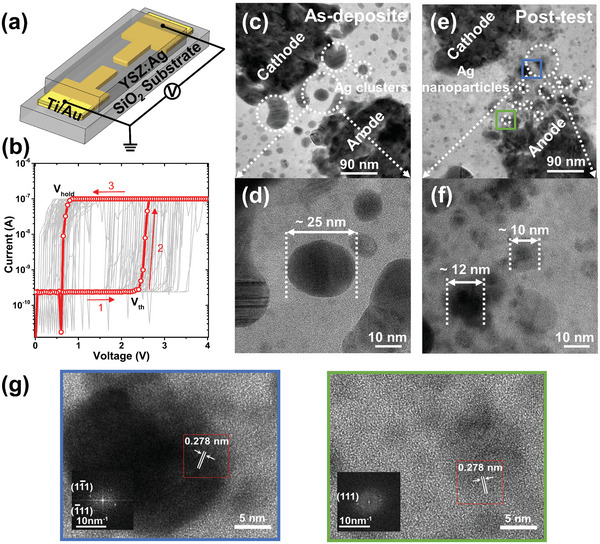
Electrical characteristics of threshold switching and microstructural evolution of an Ti/Au/YSZ:Ag/Ti/Au planar junction. a) Schematic illustration of the planar structure device, the Ti/Au electrode is 2 nm/20 nm thick. The YSZ with clustered Ag nanoparticles within the gap was the active switching region under bias. b) The DC current–voltage curves of the device in 89 consecutive positive DC sweeps. When the applied voltage crossed a threshold (*V*_th_) ≈2.5 V, the device switched to the LRS. Once the voltage is below a hold voltage (*V*_hold_) ≈0.5 V, the device switched back to the HRS. The threshold and hold voltages of the device were both evolving. c–f) TEM observation of the threshold switching process. The as‐deposit device featured large Ag clusters of ≈25 nm in (c) and (d). DC sweeps broke Ag clusters into small Ag nanoparticles of ≈10 nm in (e) and (f), forming a cone shape percolation path. g) HRTEM of the blue and green box region in (e). The insets are fast Fourier transforms of the red boxes, indicating the crystalline nature of Ag nanoparticles.

In order to unravel the underlying switching mechanism of the observed second‐order memristive dynamics, the bias‐history dependent delay and relaxation, a planar structured Ti/Au/YSZ:Ag/Ti/Au memristor with 2 nm/20 nm thick Ti/Au electrodes and a nanojunction was fabricated for TEM inspection as shown in **Figure** **2**a.^[^ ^57^ ^]^ The TEM samples shared the identical metal–insulator–metal junction with that of vertically stacked memristors. The planar memristor shared similar electrical characteristics with the Ti/Au/YSZ:Ag/Au crossbar memristor. The DC *I*–*V* characteristics of the device under positive sweeping voltages was shown in Figure 2b. Like that of the crossbar memristor, when the applied voltage crossed a threshold (*V*_th_) ≈2.5 V, the device switched to its LRS. Once the bias dropped below a hold voltage (*V*_hold_) ≈0.5 V, the device switched back to the HRS from LRS. The observed repeatable cyclic threshold switching between the two states, with an increased operating voltage due to a larger separation between electrodes, indicated that the crossbar and planar memristors possess the same switching mechanism. The threshold and hold voltages of the device were both dynamic, spanning an increased interval ≈2 and ≈1 V, respectively. The memristor withstood a reduced ON‐state current of 100 nA. The device exhibited decent repeatability under consecutive DC voltage sweeps, as no visible deterioration was observed (see Figure 2b where a representative switching cycle was labelled in red). Therefore, the planar YSZ:Ag memristor provided a credible platform to probe the switching mechanism of the crossbar memristors where the active switching regions were not easily accessible.

The evolution of the device microstructures was observed in Figure 2c,e (more microscopic observations at different magnifications are with Figure S2 in the Supporting Information), which shows the nanogaps between the two electrodes in an as‐deposited planar memristor and the same device after electrical operations. These TEM images are consistent with the early observation that the device operates on the formation and rupture of metallic filaments due to the underlying redox reactions and cation transport.^[^ ^68^, ^69^ ^]^ Specifically, when a constant positive voltage was applied across the nanogap, Ag atoms, such as those with the positively biased electrode, will be oxidized to Ag^+^ cations. Assisted by local temperature rise due to Joule heating and the external electric field, the Ag^+^ cations will diffuse and drift before being reduced and nucleated on the downstream adjacent Ag particles or electrode, the so‐called bipolar electrode effects.^[^ ^68^, ^70^ ^]^ Such a process will significantly impact the distribution of Ag in the nanogap, which may be critical for the observed second‐order memristive dynamics. For example, large Ag clusters of ≈25 nm in the as‐deposited memristor (Figure 2d) broke into a collection of smaller nanocrystalline Ag particles of ≈10 nm after receiving the electrical stimulus (Figure 2f). The observed morphological changes in Figure 2e may be explained as follows. The filament growth first took place on the anode side. Given the sputtered YSZ was a poor electrolyte, the Ag^+^ ion mobility and redox rate are limited. During subsequent growth, the small amount of Ag^+^ ions might migrate over a short distance within the dielectric film before they were reduced by capturing incoming free electrons. Such reduction is likely to occur at the edge of the existing filament, because of the geometric enhancement of the electric field by the filamentary protrusion. In this case, the Ag precipitates were formed near the anode and will serve as a patch for its expansion. This repeated ionization, short‐distance transport and reduction process eventually led to filament growth from the anode towards the cathode during cyclic programming with positive DC sweeps. As a result, the nanogap region interfacing with the cathode had the narrowest width in a forward cone filled with Ag nanoparticles, or filamentary residuals, in Figure 2e and thus would be most critical during device operations. Since the size of Ag nanoparticles and their separation gradually reduced over time, this results in the observed reduction of switching delay time, which contrasted to the conventional electrochemical metallization (ECM) model for solid electrolytes^[^ ^71^, ^72^ ^]^ and the valence change model due to the redistribution of anions.^[^ ^58^, ^65^, ^73^, ^74^ ^]^

Upon the cease of applied voltage, the Ag filament incurred additional surface energy compared to that of Ag nanoparticles, generating extra chemical potential gradients according to the Gibbs–Thomson effect, starting to coalesce. This led to surface diffusion of Ag atoms towards their minimum energy positions,^[^ ^75^ ^]^ assisted by the residual heat, upon the cessation of the electrical power. In addition, the dissolution of the filament was also influenced by the observed Nernst potential, the diffusion potential, together with the Gibbs–Thomson effect, which constitutes the nanobattery effect.^[^ ^47^, ^53^, ^76^, ^77^ ^]^ Eventually, the Ag filament broke into a series of Ag nanoparticles separated by gaps at nanometer scale (see Figure 2e,f). The crystalline nature of these Ag nanoparticles was confirmed by high‐resolution TEM images (see Figure S3 in the Supporting Information) along with the fast Fourier transfer (FFT) pattern, as shown in Figure 2g, where the lattice fringes were attributed to the (111) plane of Ag. The residual Ag conductive filament was not completely bridging the two electrodes over the dielectric, which accords with the experimental observation that the HRS was reinstated after the removal of the switching voltage. It is worth noting that the final residence of Ag nanoparticles depended on the interfacial energy between Ag and the host dielectric, mobility of Ag atoms in the host matrix, the thinness of the dielectric layer and the ambient temperature. In case of a relatively small interfacial energy between Ag and the host dielectric, low mobility, thick dielectric layer, and fast temperature decay, the Ag atoms might only be able to migrate to local energy‐minimal positions within the dielectric layer rather than a global energy minimization configuration of the system, leading to nanoparticles of smaller dimensions. In addition, after the initial rupture of the filament at the narrowest region near the filament/cathode interface, the rest of the filament was no longer electrically connected to the cathode under the positive bias, resulting in less‐efficient redox processes. As a result, a substantial portion of filament still remained intact, which could facilitate subsequent regrowth of filament and impede its rupture. This is especially significant if the interfacial energy between Ag and host dielectric (e.g. YSZ) is relatively small, so repetitive pulses led to the accumulation of small Ag nanoparticles within the nanogap and a gradual increase in switching speed, consistent with the observation shown in Figure 1d. (see more evidence on the threshold switching and STL mechanisms in Note S1 of the Supporting Information) This second‐order memristive dynamics property, bearing a strong resemblance with that of synaptic and neuron plasticity, in particular the STL, was revealed for the first time using TEM in electrochemical metallization cells and provided insights for the design of higher‐order volatile memristors.

**Figure 3:**
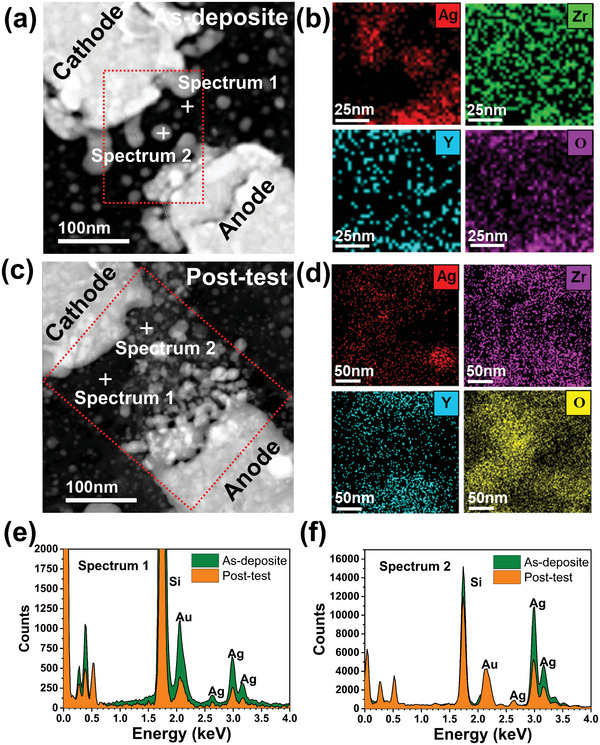
Energy‐dispersive X‐ray spectroscopy (EDS) analysis of the Ti/Au/YSZ:Ag/Ti/Au planar junction memristor. a,c) Scanning transmission electron microscopy (STEM) images of the as‐deposited and post‐tested junction gap region where the Ag clusters and nanoparticles are located. b,d) EDS elemental mapping of Ag, Zr, Y, and O of the region highlighted by the red dash box in (a) and (c), respectively. e,f) EDS point spectrums of the corresponding cross label marks in (a) and (c), respectively.

The chemical information of the observed filaments, such as their compositions and chemical states, were also examined through analytical TEM. The scanning transmission electron microscopy (STEM) images and energy dispersive X‐ray spectroscopy (EDS) elemental maps revealed the spatial distribution of elements in the device. The bright field STEM images clearly show that the large Ag clusters (white round‐shape spots in junction gap) in **Figure** **3**a of the as‐deposited device broke into smaller Ag nanoparticles that are distributed uniformly between the top electrode and bottom electrode, forming a cone shape Ag nanoparticle percolation path after electrical operations as shown in Figure 3c. Compared to the Ag elemental mapping of the as‐deposited memristor in Figure 3b, there was no observation of any obvious aggregation of Ag elements at the same gap location in the post‐test device. Instead, the Ag was more evenly distributed within the junction gap according to the Ag mapping in Figure 3d, consistent with the aforementioned switching mechanism. Different from the redistribution of Ag element, the distributions of Zr and Y elements remain unchanged (see Figure S4 in the Supporting Information for other elemental mapping). They were uniformly distributed in both as‐deposited and post‐tested devices, suggesting both Zr and Y were immobile backbones of the dielectric host matrix. It is also observed that the distribution patterns of O and Ag were similar. A possible explanation is that the introduction of Y^3+^ dopants into the ZrO_2_ matrix created a large number of oxygen vacancies according to the reaction Y→Y′_Zr_+1/2V_O_. When a positive bias voltage was applied, the electrochemical reactions between Ag^+^ and O vacancies might lead to the formation of silver oxide, as such the Ag and O element would share similar elemental distributions. However, further experiment is needed to justify this hypothesis. To precisely probe the composition of the Ag nanoparticles and background dielectric, EDS point spectrum analysis was carried out. The spectrum analysis confirmed the atomic and weight fractions of Ag, Zr, Y as summarized in Tables S1 and S2 in the Supporting Information. Comparing the spectrum 1 and 2 in Figure 3e,f (see more point spectrums in Figure S5 in the Supporting Information), the counts of Ag atoms significantly reduced after the cyclic programming, consistent with the proposed switching mechanism.

### 2.3 SCNN with Spatial Attention for Multiobject Detection

**Figure 4:**
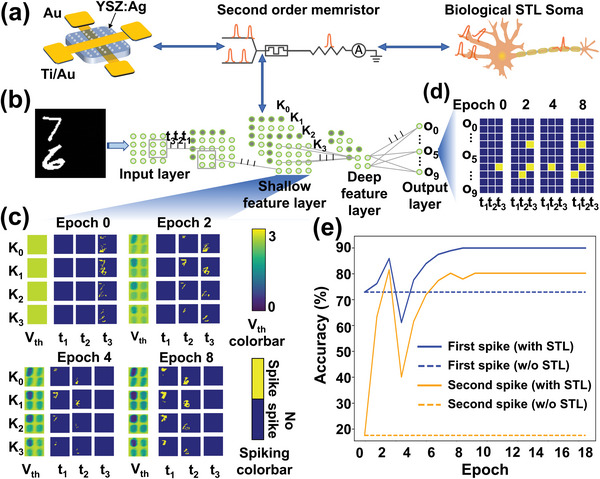
Second‐order YSZ:Ag memristors‐based spontaneous threshold lowering (STL) neurons for spatial attention in a spiking convolutional neural network (SCNN). a) YSZ:Ag second‐order memristors were used to implement the STL neurons. b) The architecture of the SCNN. The shallow feature layer consists of four channels (K_0_, K_1_, K_2_, and K_3_) and the output layer consists of ten nodes (O_0_ to O_9_). See Notes S4 and S5 for details. c) Threshold potential maps of the feature layer neurons (left column in each epoch) and spiking maps of the four channels of feature layer neurons (rest columns in each epoch) at epoch 0, 2, 4, and 8. Here each inference propagation is divided into three time steps, *t*_1_, *t*_2_, and *t*_3_. d) The spiking maps of the output layer neurons at epoch 0, 2, 4, and 8. e) The classification accuracy of the first spike (for the object within the area of interest) and the second spike (for the object outside the area of interest) without (dashed line)/with (solid line) STL neuron.

The human visual system features a spatial attention mechanism that can deal with a large receptive field with limited neurons.^[^ ^78^, ^79^ ^]^ The brain cortex, effectively functioning as an object detector receiving a subset of the receptive field, pays attention to individual objects in complex backgrounds regardless of their spatial locations.^[^ ^79^ ^]^ The key to this spatial attention is the STL vision neurons (see Note S2 in the Supporting Information for details on STL and spatial attention), which can be effectively simulated using our second‐order YSZ:Ag memristors. Since the resistive switching threshold (or SET voltage) of the memristor can be lowered by incoming voltage spikes, this effectively replicates the lowering of the threshold potential of the soma, as shown in **Figure** **4**a. Here we simulate a memristor‐based SCNN with spatial attention shown in Figure 4b using two energy‐efficient coding schemes, the spiking regulation scheme that a shallow feature neuron spike at most once in an inference propagation and the lateral inhibition scheme that resets all the output neuron membrane potential if any output neuron spikes (see Note S4 in the Supporting Information for the details of the SCNN with self‐adaptive spatial attention). We consider the case where multiple objects (e.g., handwritten digits) simultaneously appear in different corners of the receptive fields, as shown in Figure 4b left. These objects appear in different corners of the receptive field at different frequencies (e.g., one of the two objects always appears in the left‐up corner of the square receptive field, see Figure S6 in the Supporting Information for the examples of the dataset). The part of the receptive field where the object appears frequently is the area of interest. The synaptic connections (weights) of the spatial attention‐based SCNN model have been preoptimized and stay fixed during the course of inference (see Note S3 in the Supporting Information for the optimization of the synaptic weights of the SCNN). Figure 4c shows the threshold map and the spike map of the shallow feature layer at different inference epochs. At the beginning of the inference (i.e., epoch 0), the thresholds of all neurons in the shallow feature layers are the same, thus neurons responding to different parts of the receptive field (corresponding to different letters) spike at the same time (i.e., all neurons spike at *t*_3_), as shown in the spiking feature map (in blue/yellow color map) of Figure 4c. This makes the downstream object detector less capable to recognize different objects, known as the “binding problem”, because the simultaneous presence of features at different spatial locations confuses the downstream object detector.^[^ ^80^ ^]^ Subsequently, in epoch 2, 4, and 8, the threshold of memristor‐based visual neurons start to decrease due to inherent STL. Those within the area of interest (e.g., upper left area) decrease more than that of the rest, inducing earlier spiking of neurons in this part of the receptive field once stimulated. For instance, at epoch 2, the feature layer neurons in the area of interest have lower thresholds, thus spiking at time spike *t*_2_. On the contrary, the feature layer neurons outside the area of interest are with a relatively higher threshold, thus spiking at *t*_3_. This results in temporal separation of features from different spatial locations, or the spatial attention that focuses on the area of interest in a self‐adaptive manner, which overcomes the “binding problem” in the traditional SCNN (see Note S5 in the Supporting Information for SCNN internal state analysis). As shown in Figure 4d, the corresponding output spikes of the downstream object detector tend to appear in a shorter time compared to the system with fixed threshold visual neurons, for example, at epoch 8, the object detector first spike at *t*_1_ followed by spiking at *t*_2_, thanks to the STL mechanism of neurons, which also saves energy and time. In Figure 4e, solid lines show the accuracy of the multiobject classification using STL neurons, which is improved from 70% to 90% for the object in the area of interest (first spike) and from 20% to 80% for the objects outside the area of interest (second spike). In comparison, the dashed lines show the accuracy of SCNN without STL neurons, which maintains the same threshold for the entire inference period, resulting in a constant low accuracy from Epoch 0. The ablation experiment without STL neurons is discussed in detail in Note S7 in the Supporting Information.

**Figure 5:**
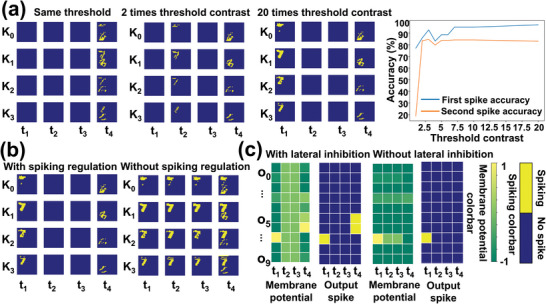
Impact of threshold contrast (defined as the ratio between the original and the lowered threshold of the memristors‐based STL neuron), spiking regulation (each neuron spikes at most once per input sample), and lateral inhibition (firing of a neuron resets the membrane potentials of all other neurons in the same layer). a) SCNN shallow feature layer spiking maps under different threshold contrasts and the corresponding classification accuracy. A larger threshold contrast leads to a larger temporal separation of features from different objects, benefiting multiobject classification. b) SCNN shallow feature layer spiking maps with (left)/without (right) spiking regulation. Spiking regulation helps to eliminate the influence of early spiking features over the later ones, improving classification accuracy and reducing inference energy. c) SCNN output layer membrane potential map and spiking map with (left)/without (right) lateral inhibition scheme. Lateral inhibition scheme helps to eliminate the temporal correlation of features at different time step, improving classification accuracy.

We further investigate the impact of STL, specifically the threshold contrast (defined as the ratio between the threshold of the memristors‐based STL neurons that are outside and within the area of the interest), on spatial attention performance. **Figure** **5**a shows the feature layer spiking maps of the SCNN under different threshold contrasts, where the threshold contrasts ranging from 2 to 20 lead to a clear temporal separation of features and a significant accuracy increment, as shown in the right part of Figure 5a. Notably, a larger threshold contrast leads to a larger temporal separation, which may be more promising for multiobject classification. The decreasing rate of the threshold affects the number of epochs in realizing spatial attention, but makes no difference in the eventual classification accuracy (see Figure S7 in the Supporting Information). We also investigate the advantage of spiking regulation and lateral inhibition. First, we evaluate the SCNN with spiking regulation (i.e., each neuron spikes at most once per input sample) in comparison to the one without spiking regulation. As shown by the feature layer spiking maps in Figure 5b, the spiking regulation reduces the number of spikes for inference and avoids the influence of early feature (e.g., the feature corresponding to the digit 7) on the later feature (e.g., the feature corresponding to the digit 6). In addition, Figure 5c shows the effect of lateral inhibition (i.e., firing of a neuron resets the membrane potentials of all other neurons in the same layer.) of the SCNN output layer in comparison with the one without lateral inhibition. The lateral inhibition helps to produce temporally independent classification results at each time step based on current spiking feature maps. Moreover, the YSZ:Ag memristor‐based neuron is relatively robust for the spatial attention although there is inevitable stochasticity in the underlying electrochemical reactions (see Note S6 in the Supporting Information for detailed reliability and robustness analysis). As such, the second‐order YSZ:Ag memristor‐based STL neurons, paired with the spiking regulation and lateral inhibition, not only improve multiobject classification accuracy thanks to the physically encoded spatial attention but also improves the speed and efficiency of the SCNN by leveraging the spiking regulation and lateral inhibition.

## 3 Conclusion

In summary, inspired by the neuronal excitability modulation of biological neurons, we demonstrated an STL neuron using second‐order memristors for self‐adaptive spatial attention. We first showed the existence of the second state variable in the YSZ:Ag‐based memristor and explored its physical origin via high‐resolution TEM and EDS analysis. Then, we simulated the SCNN made of YSZ:Ag memristor‐based STL neurons for multiobject detection, which leverages the STL to form spatial attention on the area of interest. The resultant temporal separation of features of different objects improves classification accuracy on multiple objects in the receptive field as well as the system energy efficiency and speed. Such second‐order memristors not only overcome the scaling and von Neumann bottlenecks of CMOS digital hardware but also possess rich and bio‐plausible dynamics for future machine intelligence.

## 4 Experimental Section

### Sample Preparation

YSZ:Ag‐based Memristor Device Fabrication—For the crossbar‐structured diffusive memristor, a p‐type (100) Si wafer with 100 nm thermal oxide was used as the substrate. The standard photolithography and lift‐off process were used to define the device size, followed by electron beam evaporation of Ti (2 nm)/Au (20 nm) bottom electrodes. Then a 10 nm thick blanket YSZ:Ag switching layer was deposited by RF cosputtering in Ar and O_2_ mixed ambient at room temperature using a YSZ target and Ag target. Again, the standard photolithography and lift‐off process were applied to the 20 nm top Au electrodes which were deposited by electron beam evaporation. Thanks to the good adhesion between the top Au layer and Ag particles scattered within the YSZ:Ag layer, no extra adhesive metal interlayer was introduced. The nanogap planar device shared the same substrate, bottom electrodes and dielectric layer as the vertical diffusive memristor except a second layer of dielectrics was then deposited with an increased thickness of ≈20 nm after the metal electrodes.

### Characterization

Electrical measurements—Electrical measurements were performed with a Keysight B1500A semiconductor parameter analyzer using two of its modules, DC measurements were carried out using the source and measure units (B1517A) and the B1530A waveform generator/fast measurement unit (WGFMU) was used to perform the pulsed measurements. Using a two‐probe (W tips) configuration, DC and pulse voltages were applied between the top (anode) and bottom (cathode) electrodes of the memristor and measured current through one of the measurement units. For each data point of the cyclic programming measurement, the crossbar memristor was in its high‐resistance state at first, and then a voltage pulse was applied across memristor in each cycle with the same pulse amplitude (1.5 V), duration (1 ms), and finally read the corresponding current response across the device to determine the change in its dynamic performance induced by the pulse.

Transmission Electron Microscopy—High resolution scanning transmission electron microscopy (STEM)/dispersive X‐ray spectroscopy (EDS) analysis was performed with a FEI Titan TEM at an accelerating voltage of 300 kV.

### Spatial Attention based SCNN Implementation

Stage 1: SCNN preoptimization. The convolutional kernels and object detector (i.e., dense layer) were first optimized by the surrogate gradient descent in the PyTorch framework (see Note S1 in the Supporting Information).^[^ ^81^ ^]^

Stage 2: SCNN inferencing with STL neurons. The SCNN consists of the preoptimized convolutional kernels and object detector with STL neurons (see Note S2 in the Supporting Information). The forward inference was carried out on the multiobject dataset (see Figure S6 in the Supporting Information), where the threshold potential of neurons decreases adaptively to form spatial attention.

## Conflict of Interest

The authors declare no conflict of interest.

## Supporting information

Supporting Information

## Data Availability

The data that support the findings of this study are available from the corresponding author upon reasonable request.
