# Supplementary material for: Spontaneous Threshold Lowering Neuron using Second‐Order Diffusive Memristor for Self‐Adaptive Spatial Attention
Source: Adv Sci (Weinh). 2023 May 24;10(22):2301323. doi: 10.1002/advs.202301323 (PMC10401116; doi:10.1002/advs.202301323)
Supplement: Supplementary file 1 — Supporting Information [file ADVS-10-2301323-s001.pdf]

ADVANCED SCIENCE
Open Access

Supporting Information

for *Adv. Sci.*, DOI 10.1002/advs.202301323

Spontaneous Threshold Lowering Neuron using Second-Order Diffusive Memristor for Self-Adaptive Spatial Attention

Yang Jiang, Dingchen Wang, Ning Lin, Shuhui Shi, Yi Zhang, Shaocong Wang, Xi Chen, Hegan Chen, Yinan Lin, Kam Chi Loong, Jia Chen, Yida Li, Renrui Fang, Dashan Shang, Qing Wang*, Hongyu Yu* and Zhongrui Wang**

Supporting Information

Spontaneous Threshold Lowering Neuron using Second-order Diffusive Memristor for Self-adaptive Spatial Attention

Yang Jiang†, Dingchen Wang†, Ning Lin, Shuhui Shi, Yi Zhang, Shaocong Wang, Xi Chen, Hegan Chen, Yinan Lin, Kam Chi Loong, Jia Chen, Yida Li, Renrui Fang, Dashan Shang, Qing Wang*, Hongyu Yu*, Zhongrui Wang**

† These authors contributed equally.

Y. Jiang, D. Wang, N. Lin, S. Shi, Y. Zhang, S. Wang, X. Chen, H. Chen, Y. Lin, K. C. Loong, Prof. Z. Wang
Department of Electrical and Electronic Engineering, The University of Hong Kong, Pokfulam Road, Hong Kong
E-mail: zrwang@eee.hku.hk

Y. Jiang, D. Wang, N. Lin, S. Shi, Y. Zhang, S. Wang, X. Chen, H. Chen, Y. Lin, K. C. Loong, Dr. J. Chen, Prof. Z. Wang
ACCESS – AI Chip Center for Emerging Smart Systems, InnoHK Centers, Hong Kong Science Park, Hong Kong

Y. Jiang, Y. Zhang, S. Shi, Prof. Y. Li, Prof. Q. Wang, Prof. H. Yu
School of Microelectronics, Southern University of Science and Technology, Shenzhen, 518055, China
Email: wangq7@sustech.edu.cn; yuhy@sustech.edu.cn

R. Fang, Prof. D. Shang
Institute of Microelectronics, Chinese Academy of Sciences, Beijing, 100029, China
Email: shangdashan@ime.ac.cn

Keywords: spontaneous threshold lowering, second-order memristor, self-adaptive spatial attention, multi-object recognition, spiking convolutional neural network

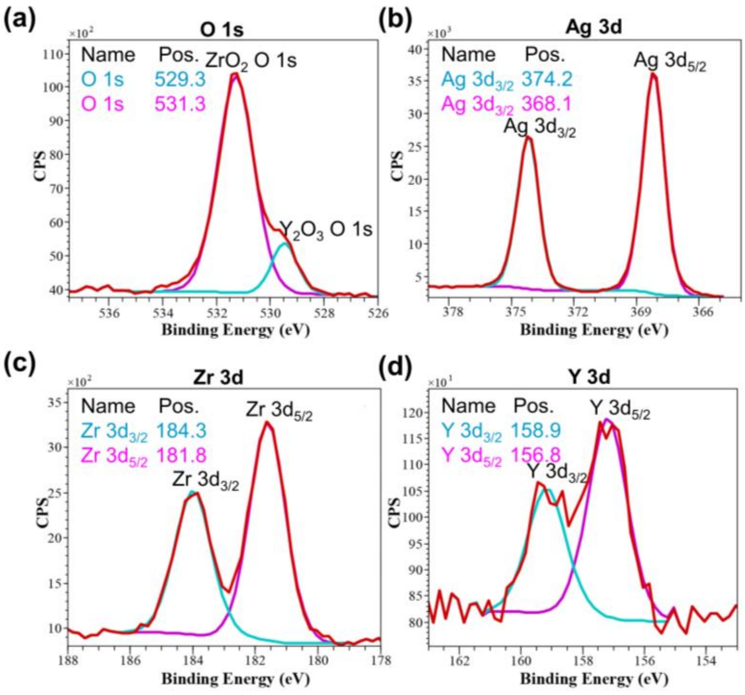

Figure S1. X-ray Photoelectron Spectroscopy (XPS) measurements along with fitting results of (a) O 1s, (b) Ag 3d, (c) Zr 3d and (d) Y 3d peaks of the YSZ:Ag switching layer. All spectrums are calibrated by aligning C 1s to 284.6 eV.

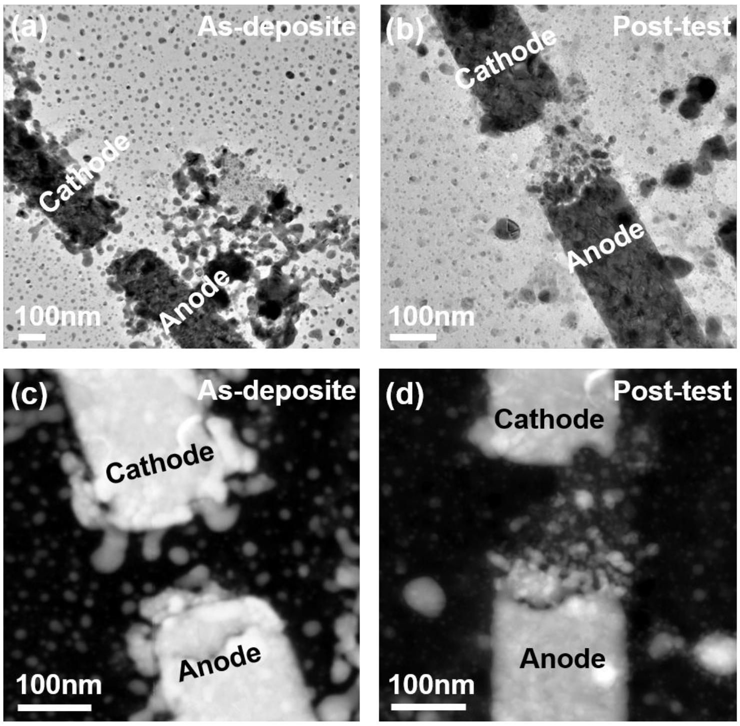

Figure S2. YSZ:Ag-based diffusive memristor undergoing resistive switching at different magnification. (a) and (b) bright field transmission electron microscopy (TEM) images of the as-deposited and post-test planar junction, respectively. (c) and (d) Scanning transmission electron microscope (STEM) high-angle annular dark-field (HADDF) images of the as-deposited and post-test planar junction, respectively.

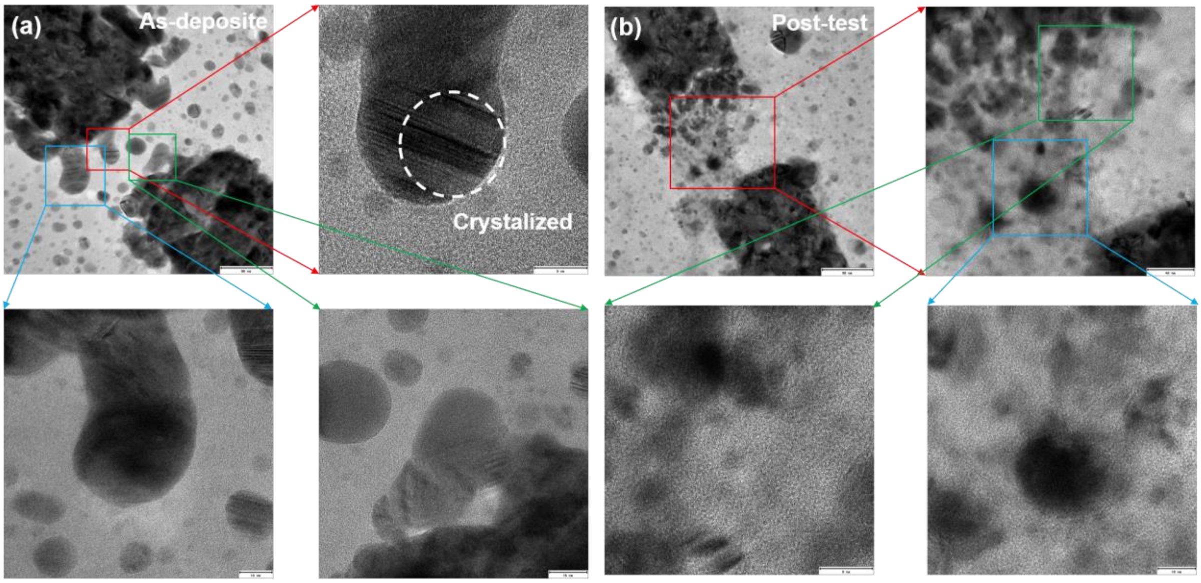

Figure S3. TEM images of the (a) as-deposited Ag clusters and (b) post-test Ag nanoparticles. The fringes of Ag clusters and nanoparticles revealed their crystalline nature.

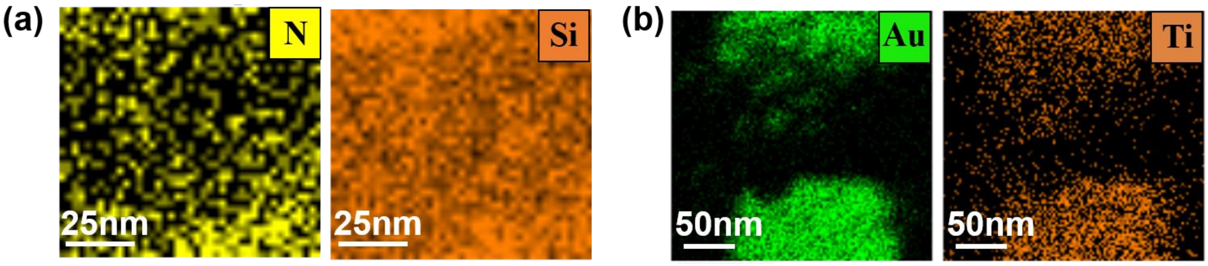

Figure S4. Energy-dispersive X-ray spectroscopy (EDS) elemental mapping of (a) N and Si of the region highlighted by the red dash box in **Figure 3a** and (b) Au and Ti of the region highlighted by the red dash box in **Figure 3d**, respectively.

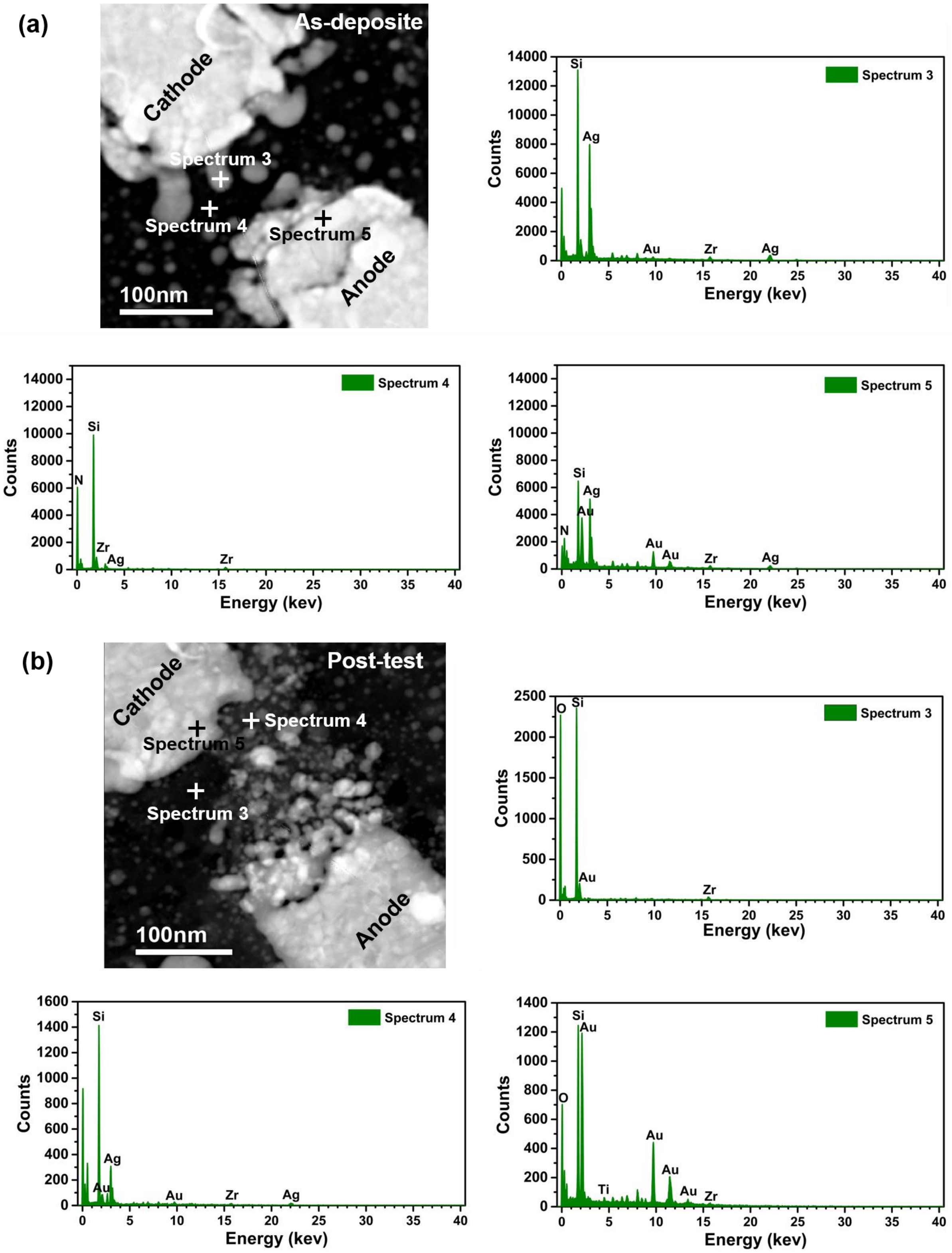

Figure S5. EDS point spectrums of as-deposited and post-test memristors. (a) STEM image of the as-deposited junction gap region where the Ag clusters are located and corresponding EDS point spectrums of the cross label marks. (b) STEM image of the post-test junction gap region where the Ag and nanoparticles are located and corresponding EDS point spectrums of the cross label marks.

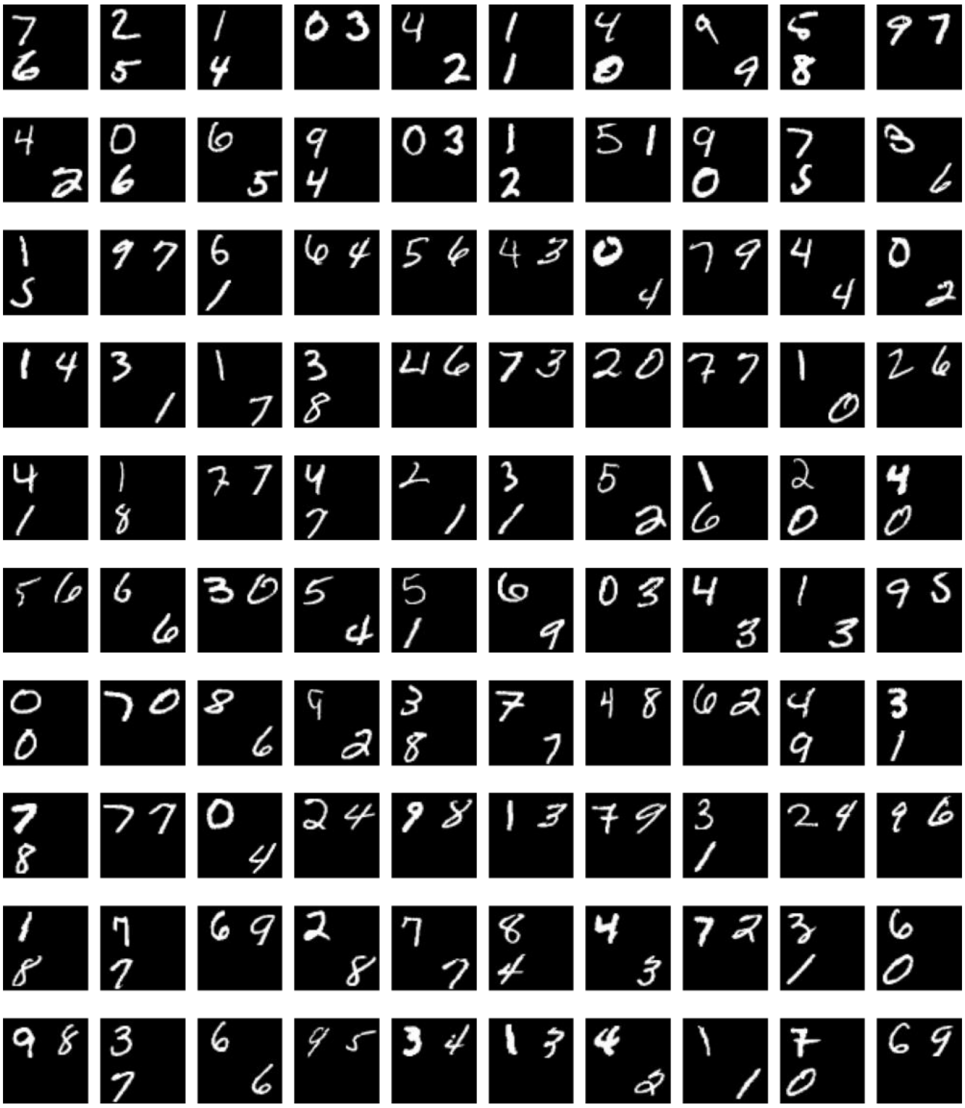

Figure S6. Illustration of synthesized dataset for multiple object recognition. Here each sample accommodates two handwritten digits at different corners. The dataset consists of 60,000 samples with 10,000 randomly choosen samples as test set.

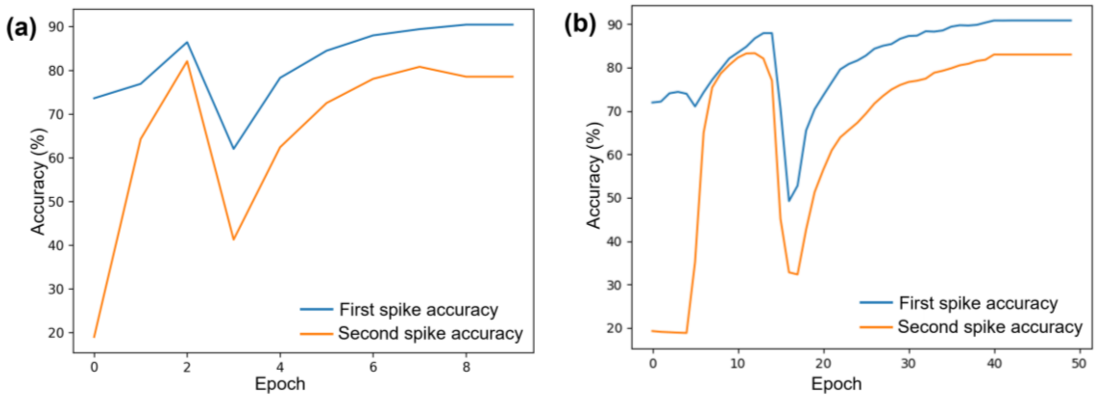

Figure S7. Impact of the threshold lowering rate on the learning performance. Accuracy of the network with STL neurons of a lowering rate (a) ~$0.697^{0.0001}$ (b) $0.697^{0.00002}$.

Table S1. Element concentrations in the as-deposited memristor according to EDS point spectrums in **Figure 3c**.

As-deposited	Spectrum 1		Spectrum 2	
Elements	Atomic fraction (%)	Weight fraction (%)	Atomic fraction (%)	Weight fraction (%)
Ag	34.15	38.08	88.70	90.30
Zr	58.54	55.20	10.17	8.76
Y	7.31	6.72	1.13	0.94

Table S2. Element concentrations in the post-test memristor according to EDS pointspectrums in **Figure 3f**.

Post-test	Spectrum 1		Spectrum 2	
Elements	Atomic fraction (%)	Weight fraction (%)	Atomic fraction (%)	Weight fraction (%)
Ag	31.90	35.66	84.14	86.25
Zr	66.01	62.41	15.64	13.56
Y	2.09	1.93	0.02	0.19

Table S3. Comparison with literature reported adaptive neuron models based on memristors.

**Ref.**	**Threshold Adaptation Mode**	**Implementation**	**Emerging Memory Used**	**Adaptation Circuit**	**Classification Task (Accuracy)**	**Neural Network (Algorithm)**	**Biological background**
[1]	Threshold lowering	Experimental	SiO_x:Ag memristor	None	-	-	Nociceptor
[2]	Threshold increasing	Simulation	RRAM	2 RRAM devices	Partial MNIST (100%)	SNN (Unsupervised STDP)	-
[3]	Threshold increasing	Experimental	Bilayer OxRAM	6T, 1 OxRAM device	1. SMNIST (96.1%) 2. Google Speech Commands (91%)	RSNN (BPTT)	Neuronal adaptation
[4]	Threshold increasing	Simulation	PCM	1T, 1 PCM device, 1 OTA, 1 pulse generator	Fashion-MNIST (97%)	SNN (Unsupervised STDP)	Homeostatic regulation
[5]	Adaptation variable threshold	Simulation	VO_2 memristor	Thermoelectric ceramics, 1 light-dependent resistor	CIFAR10 (78%)	SNN (-)	Adaptive feature of the biological retina
[6]	Threshold increasing	Experimental	Co_3O_{4-x} memristor	-	Letter block recognition (98%)	SNN (-)	-
**This work**	**Threshold lowering**	**Experimental**	**YSZ:Ag-memristor**	**None**	**Multi-object dataset (90%)**	**SCNN (STL-based**	**Intrinsic neuronal**

						**self adaptive spatial attention)**	**plasticity**

Supporting Note 1. STL is a result of the unique combination of YSZ and Ag.

1. Ag doping concentration impact on the volatility of resistive switching: We fabricated two YSZ:Ag memristors (vertical MIM, cross-bar electrodes) with varying Ag doping concentrations, as shown in **Figure S8** (sample#1 with low Ag concentration, or 6W Ag RF sputtering power in co-sputtering YSZ and Ag, and sample#2 with high Ag concentration, or 12W Ag RF sputtering power of Ag during co-sputtering). **Figure S8a** and **S8c** present SEM top views of YSZ:Ag thin films with low (sample #1) and high (sample #2) Ag doping concentrations, respectively. The Ag nano-clusters formed different spatial patterns under different doping concentrations, a manifestation of the underlying nucleation mechanism.

Electrically, sample#1 showed non-volatile resistive switching (**Figure S8b**), while sample#2 exhibited volatile switching (**Figure S8d**) in I-V sweeps. This clearly shows that an increasing Ag doping concentration in the YSZ matrix leads to the observed volatile resistive switching.

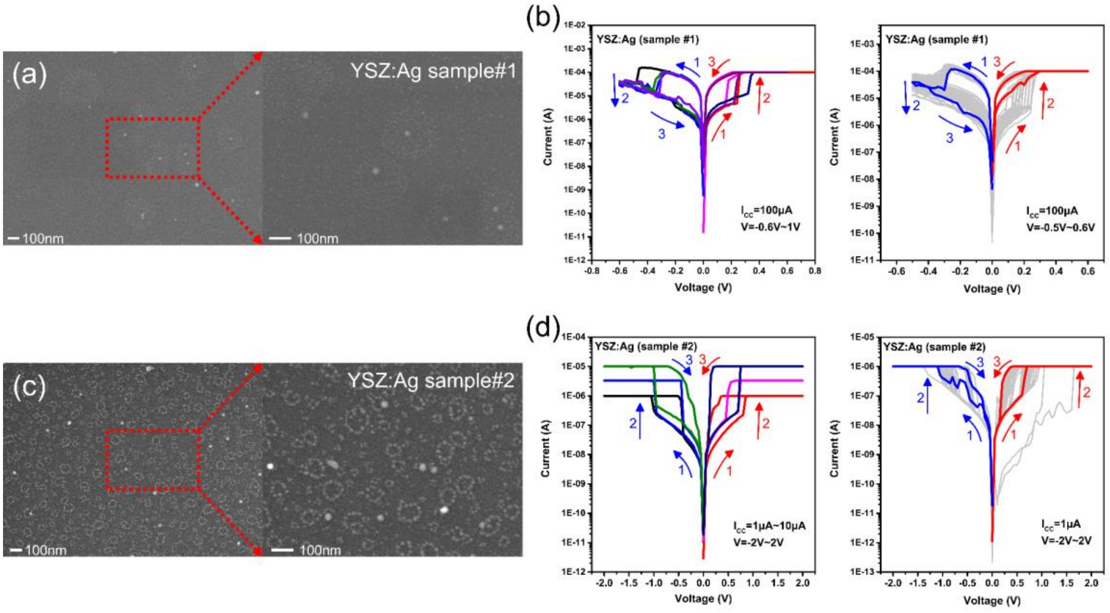

Figure S8. YSZ:Ag memristor samples with different Ag doping concentrations. (a) and (c) SEM top views of YSZ:Ag thin films with low (sample #1) and high (sample #2) Ag doping, respectively. (b) I-V sweeps of sample #1, showing non-volatile switching due to redox reaction of oxygen vacancies. (d) I-V sweeps of sample #2, showing volatile switching due to redox reaction of Ag.

2. STL due to relatively smaller interfacial energy between Ag and the host dielectric: To reveal the physical origin of the second-order dynamics and STL, we fabricated and compared SiO_x:Ag and YSZ:Ag memristors. The fabrication flow is similar to the lateral device in the main text. The Cr/Au electrodes were first defined and patterned by electron beam lithography followed by e-beam evaporation. The dielectric layers were fabricated by co-sputtering a SiO_x or YSZ target (240W) and a Ag target (12W) for 120 seconds in argon ambient.

Electrically, both SiO_x:Ag and YSZ:Ag memristors show volatile threshold resistive switching. This is consistent with the early literature where SiO_x:Ag memristor was reported as an leaky integrate-and-fire artificial neuron. However, the SiO_x:Ag memristor does not show a clear STL under repeated voltage pulsing, due to its first-order dynamics. As shown in **Figure S9a**, the switching delay was roughly a constant ~2.5μs in responding to consecutive 1.15V voltage pulses. On the other hand, for the YSZ:Ag memristor, there is a decrease in switching incubation time from 100μs to 30μs in responding to consecutive 1.5V voltage pulses (**Figure S9b**), indicating that the YSZ:Ag memristor possesses spontaneous STL behavior while the SiO_x:Ag memristor does not.

To reveal the underlying physical origin of this difference, the lateral SiO_x:Ag and YSZ:Ag memristors were inspected by SEM before and after the electrical tests, as shown in **Figure S9b and S9d**, respectively. In **Figure S9b**, it's observed that the number and size of Ag nano-clusters between the two electrodes in the SiO_x:Ag memristor did not significantly change although some clustered relocated, consistent with the observed constant switching incubation time. On the other hand, the large Ag nanoclusters were broken into smaller ones and scattered along the percolation path in the YSZ:Ag memristor after the electrical test (**Figure S9d**), consistent with **Figure 3** of the main text and the observed STL. The underlying reason between this difference is likely the interfacial energy between the SiO_x and Ag is larger than that between YSZ and Ag, therefore small Ag nanoclusters tend to be more stable in YSZ:Ag compare to that in SiO_x:Ag.

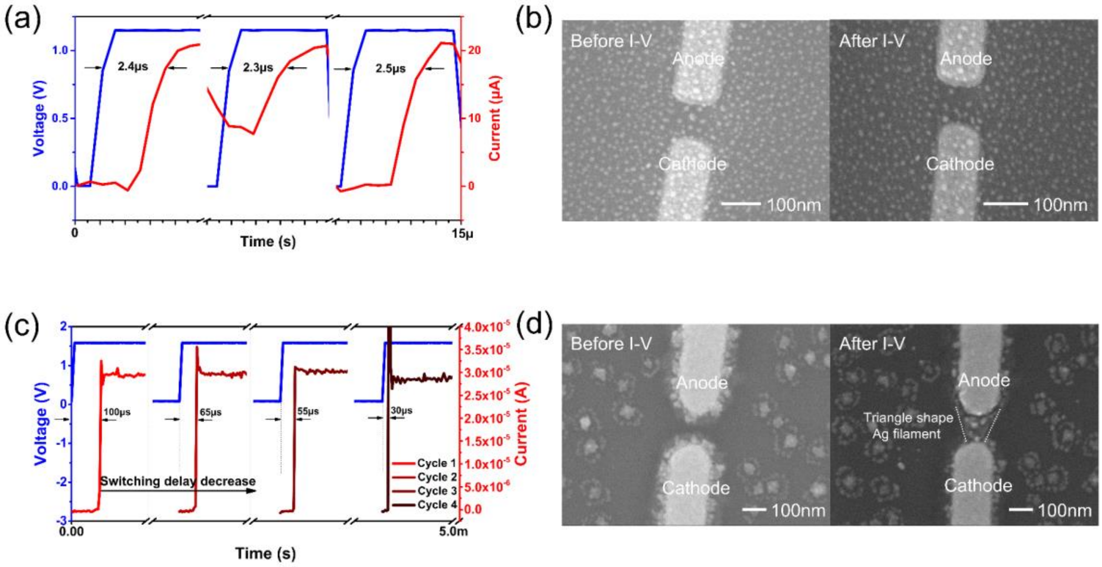

Figure S9. The comparison between SiO_x:Ag and YSZ:Ag memristors. (a) and (c) The electrical I-V pulsing tests, where SiO_x:Ag memristor showed a relatively incubation time but YSZ:Ag memristor showed a STL upon consecutive pulsing. (b) and (d) The SEM images before and after electrical pulsing test of SiO_x:Ag and YSZ:Ag memristors, respectively. The difference in the number and size of Ag nanoclusters after electrical pulsing indicates the difference in interfacial energies, which is likely the reason behind the observed STL.

The underlying mechanism of the Ag-based volatile switching has been widely studied in the prior arts, here we summarize the mechanism of YSZ:Ag based on previous discussions, which is schematically illustrated in **Figure S10**. The as-deposited Ag nano-clusters were randomly distributed in the YSZ matrix (**Figure S10a**). Upon application of a positive bias, the established electric field polarized Ag clusters and triggered electrochemical reactions. These Ag clusters acted as bipolar electrodes (BPEs), with an effective anode (δ^+) and cathode (δ^-) on opposite sides (**Figure S10b**).[7-9] Electrochemical oxidation processes generate Ag^+ ions from the anode side of the clusters, which then drift along the electric field and are reduced to Ag atoms downstream through possible reduction reactions like trapped electrons. As Ag^+ ions deposit downstream, a second Ag cluster emerges and grows, while the first cluster is slowly consumed and shrinks. When the second cluster exceeds a certain size, adequate polarization forms to oxidize the Ag atoms at its anode side, resulting in the emergence of a third cluster (**Figure S10c**).[8] This process repeats, causing a series of Ag clusters on the left to dissolve and merge into the right, leading to overall movement and redistribution of Ag clusters along the electric field direction until filaments form and bridge the electrodes.[7] Once the bias is removed, the Ag filament broke spontaneously, leading to volatile threshold switching due to the Thomson-Gibbs effect, which is caused by the surface curvature radii-related metal atoms' surface diffusion (**Figure S10d**). This effect originates

from the gradient of surface atomic vacancy concentration or the gradient of the surface atomic chemical potential, resulting in a tendency to minimize the surface energy.[11-12]

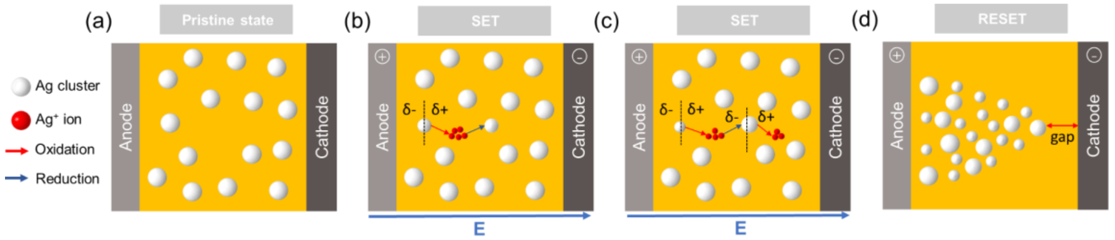

Figure S10. The schematic of Ag clusters evolution in YSZ:Ag memristor. (a) As-deposite. (b) and (c) With positive bias. (d) Bias removed.

Supporting Note 2. STL for spatial attention.

1. The biological background of spatial attention: Deep-layer neurons of the visual system feature large receptive fields, such as those located in the inferotemporal cortex.[13-14] These neurons are capable to recognize objects regardless of the spatial locations of the objects, as illustrated in **Figure S11a** and **Figure S11b**. Such location and orientation invariant object recognition is a unique feature of the brain which also contributes to the remarkable efficiency.[14]

In the presence of multiple objects, simultaneously appeared objects pose a challenge as there is a possibility of features from different objects getting mixed up, a phenomenon commonly referred to as the "binding problem".[15] This mixing of features can cause confusion to the object classifier (e.g. the inferotemporal cortex neurons), invalidate classification results, as depicted in **Figure S11b**. Therefore, to disentangle the mixed features, a spatial attention mechanism was introduced.

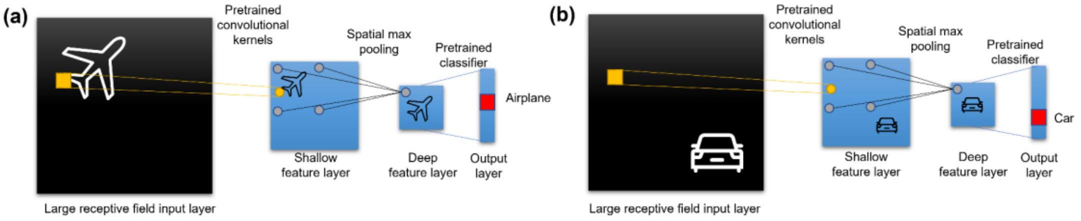

Figure S11. An increasing receptive field size allow the deep-layer neurons in visual systems to recognize an object regardless of where object's spatial location is. (a) An airplane appears at the left upper corner of the receptive field. The information is processed by pretrained convolutional kernels, spatial max pooling layers, and classified by the pretrained classifier. (b) A car appears at the right lower corner of the receptive field. The information is also classified by the same pretrained classifier.

2. "Binding problem" in spiking convolutional neuronal networks: For the spiking convolutional neuron network (SCNN) model, the "binding problem" arises when feature

layer neurons in the shallow layers spike simultaneously, resulting in the spatial and temporal mixing of features at the deep feature layer, as illustrated in **Figure S12**. Therefore, the inclusion of bio-inspired spatial attention could improve the accuracy of multiple object classification in SCNN.

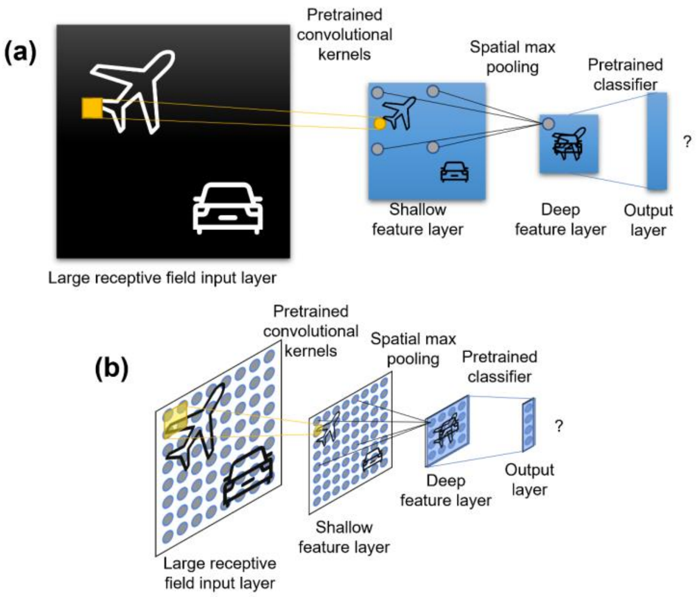

Figure S12. (a) The "binding problem" happens when multiple objects appear at the same time. The feature of multiple objects mix and confuse the single object classifier. (b) A spiking neuron network version of the "binding problem". The features of multiple objects mix spatially and temporally in deep feature layers, leading to the failure of classification.

3. How are the STL neurons used for spatial attention to solve the "binding problem": We address the binding problems by using STL neurons in the shallow feature layers to implement spatial attention mechanism, while keeping the SCNN architecture (the topology and the weights of the pretrained convolutional kernels and the single object classifiers). **Figure S13** illustrates how STL neurons, or the YSZ:Ag second-order memristors, in the shallow feature layer can practice the spatial attention mechanism in a self-adaptive manner. **Figure S13a** illustrates the initial state, where all the STL neurons have the same threshold, which results in features of different objects appearing simultaneously in the shallow layer and the "binding problem". **Figure S13b** illustrates samples of the multi-object dataset. The network is supposed to pay more attention to the region where objects appear at high frequencies, or the area of interest. Here, the area of interest is assumed to be the upper left corner. During the inference, the STL neurons in the area of interest spike more frequently, resulting in a lower threshold. Therefore, they spike at an earlier time step (t_1) and propagate features to deeper layers towards classification, as shown in **Figure S13c**. On the contrary, the STL neurons outside of the area of interest spike less frequently, leading to a relatively higher

threshold than that of neurons inside the area of interest. Thus, neurons outside the area of interest tend to spike at a later time step (t_2) as shown in **Figure S13d**. This mechanism efficiently detects multiple objects in a self-adaptive manner. A similar idea is also demonstrated in Ref [16], where different neuron thresholds lead to a different firing sequence, which is consequently used for rank encoding to classify the object in the area of interest.

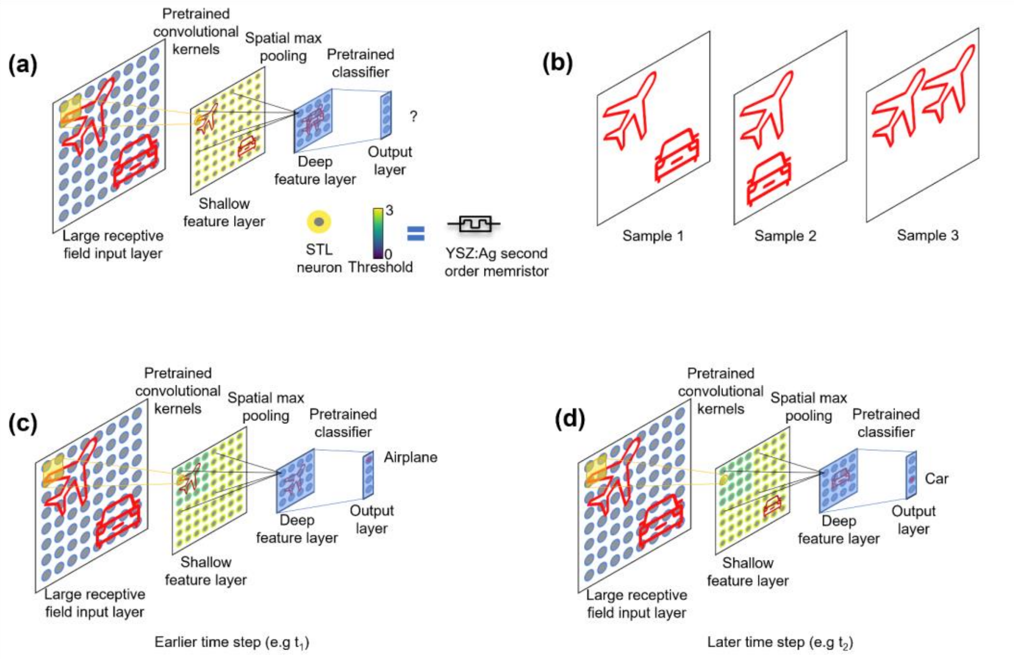

Figure S13. YSZ:Ag second order memristors-based STL neurons in the shallow feature layer, implementing spatial attention in a self-adaptive manner. (a) At first, the STL neurons have the same threshold. The features in the shallow feature layer appear simultaneously, causing the "binding problems". (b) Multiple objects appear at different spatial locations at different frequencies. Objects appear more frequently at the upper left corner, or the area of interest. (c) During inference, the STL neurons in the area of interest (upper left corner) spike more frequently, thus the threshold is lower. Consequently, they spike at an earlier time (e.g. t_1) and propagate features to deeper layer towards classification. (d) The STL neurons outside of the area of interest spike less frequently, thus the threshold is relatively higher than that of neurons within the area of interest. Thus, neurons outside of the area of interest tend to spike at a later time (e.g. t_2).

Supporting Note 3. SCNN pre-optimization.

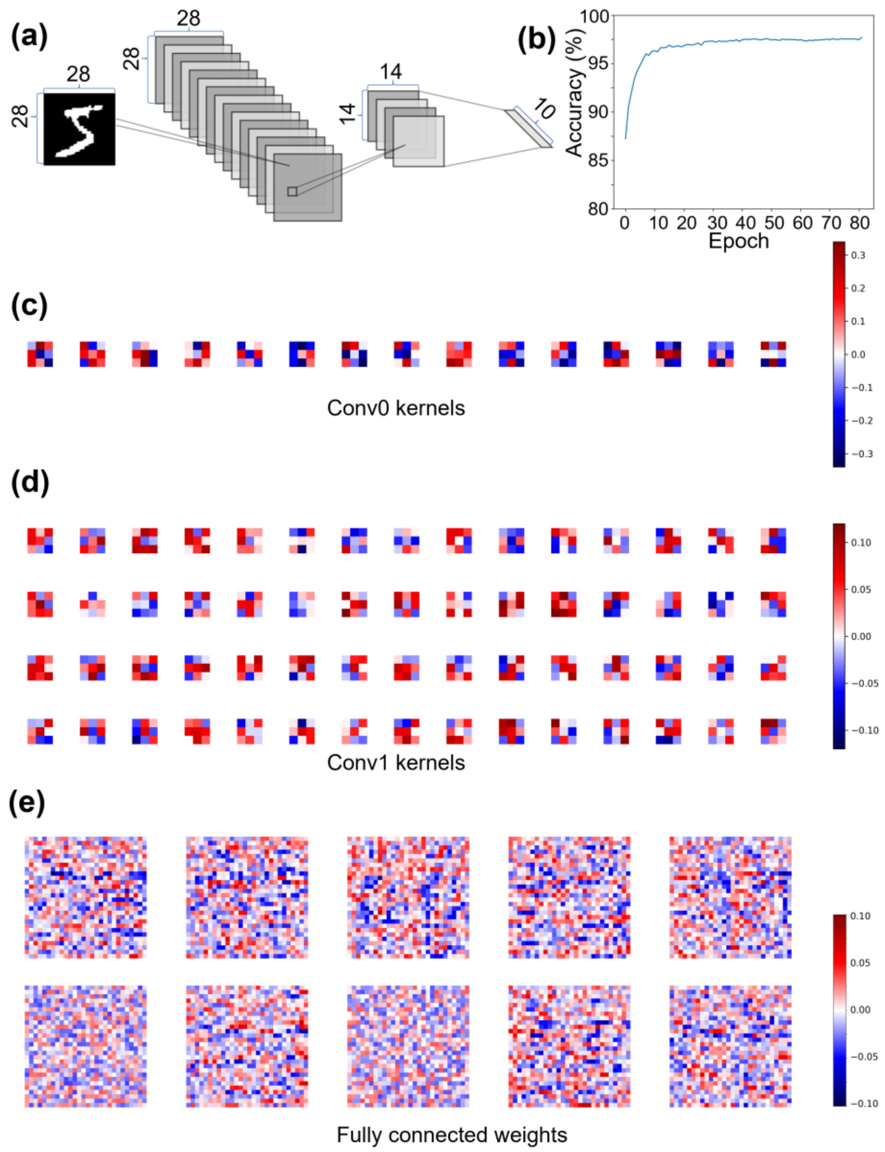

Figure S14. Pre-optimized spiking convolutional neural network (SCNN) with a small visual field for single object classification, i.e. modified national institute of standards and technology (MNIST) handwritten digits. (a) The pre-optimized SCNN consists of two convolutional layers and a dense layer. (b) The pre-optimization accuracy. (c) Weight maps of kernels of the first convolutional layer (Conv0). (d) Weight maps of kernels of the second convolutional layer (Conv1). (e) Weight maps of the fully connected dense layer.

To pre-optimize a spiking convolutional neural network (SCNN) for single object classification (i.e. modified national institute of standards and technology, MNIST, handwritten digits), surrogate gradient-based training was employed in PyTorch.[17] **Figure S14a** shows the pre-optimized model, consisting of the input layer (28×28 nodes), the first convolutional layer (15 kernels, zero padding and unit stride, or 28×28×15 nodes), the second convolutional layer (4 kernels, zero padding and unit stride followed by averaging pooling, or

14×14×4 nodes), a dense layer (10 nodes). The training is implemented via SNN backprop, a variation of backpropagation commonly used for SNNs.[17-18] The accuracy during the course of training is shown in **Figure S14b**, reaching 97% accuracy at the end. The pre-optimized kernels in convolutional layers and weights of dense layers are shown in **Figure S14c-e**. The pre-optimized kernels and dense layer weights are saved and used in the inference of SCNN with STL neurons.

Supporting Note 4. SCNN with self-adaptive spatial attention for multi-object detection.

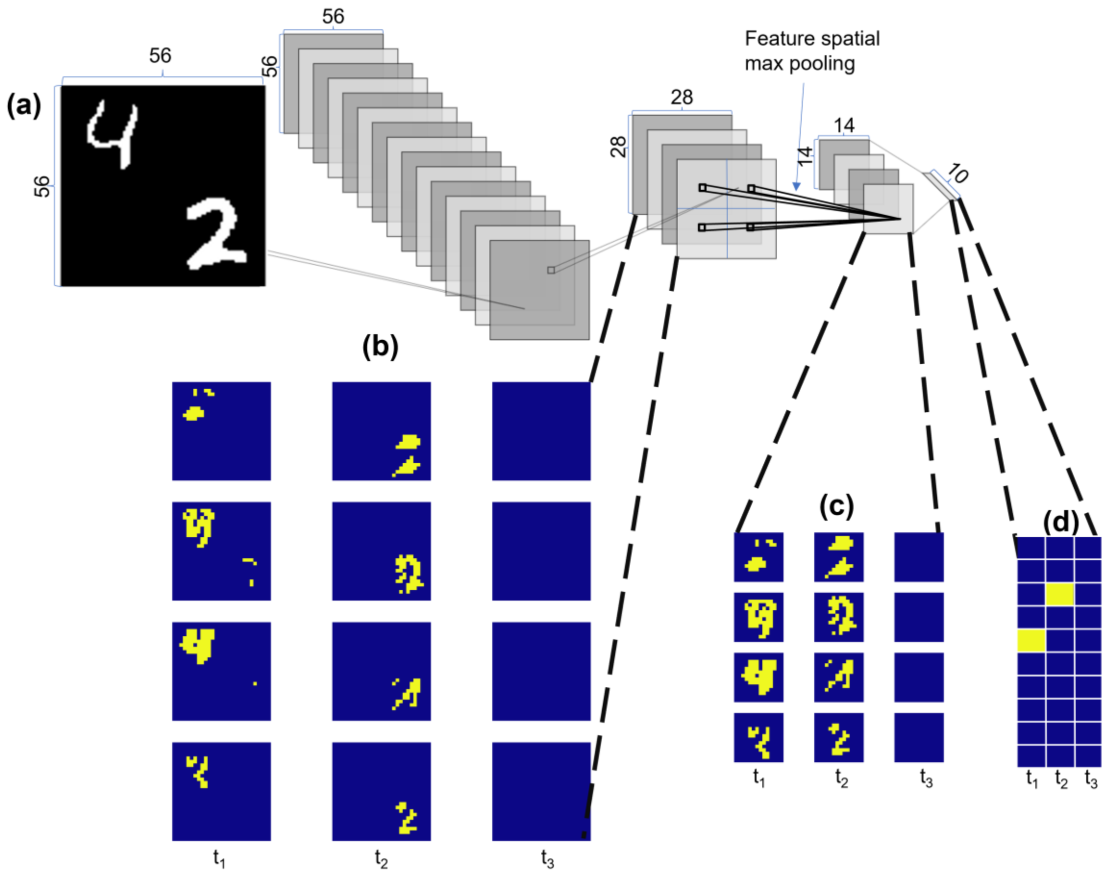

Figure S15. SCNN with self-adaptive spatial attention for multi-object detection (i.e. 56×56×1 images containing two MNIST hand written digits). (a) The SCNN consists of two pre-optimized convolutional layers, a max pooling layer, and a pre-optimized dense layer. (b) The second convolutional layer feature map by spiking neurons. (c) The max pooled spiking maps. (d) The spiking map of dense layer neurons.

Spiking Neural Networks (SNN) are bio-inspired, which employ artificial spiking neurons to model biological neuron behaviors. Famous models include leaky integrate and fire (LIF)[19], Izhikevich[20], and Hodgkin-Huxley[21]. SNN neurons encode messages through sparse and binary spikes. Neurons receive incoming spikes, these spikes are integrated and increase the

membrane potential (u) over time. Once the membrane potential reaches a threshold (V_{th}), the neuron fires an output spike (o) and resets the membrane potential. Mathematically,

$$\mathbf{u}^{t,n} = \mathbf{u}^{t-1,n}(\mathbf{1} - \mathbf{o}^{t-1,n}) + \mathbf{W}^{n} * \mathbf{o}^{t,n-1}, \tag{1}$$

$$\mathbf{o}^{t,n} = \Theta\left(\mathbf{u}^{t,n} - \boldsymbol{V_{th}}^{t,n}\right). \tag{2}$$

Where t is the time step, n is the indices of the layers of neuron of SNNs. W is the pretrained convolutional kernels. Θ(x) is a Heaviside step function which satisfies Θ(x) = 1 when x > 0, otherwise Θ(x) = 0. In addition to that, in an STL neuron, the neuron threshold (Vth) decreases by a decaying factor (d) upon each firing, according to Equation (3),

$$\boldsymbol{V_{th}}^{t+1,n} = \boldsymbol{V_{th}}^{t,n} * \boldsymbol{d}^{\mathbf{o}^{t,n}}. \tag{3}$$

If the neuron spike at time step t ($\mathbf{o}^{t,n} = \mathbf{1}$), the threshold decay by a decaying factor d ($\boldsymbol{V_{th}}^{t+1,n} = \boldsymbol{V_{th}}^{t,n} * \boldsymbol{d}^{\mathbf{1}}$). Otherwise, the threshold remains unchanged ($\boldsymbol{V_{th}}^{t+1,n} = \boldsymbol{V_{th}}^{t,n} * \boldsymbol{d}^{\mathbf{0}}$).

The SCNN with self-adaptive spatial attention mimics the human visual system, which employs a simple image classifier for multi-object detection.[16] The architecture of SCNN with spatial attention is shown in **Figure S15a**, consisting of the input layer (56×56 nodes), the first pre-optimized convolutional layer (15 kernels, zero padding and unit stride, or 56×56×15 nodes), the second pre-optimized convolutional layer (4 kernels, zero padding and unit stride follwoed by averaging pooling, or 28×28×4 nodes, the feature layer), the addtional max pooling layer which takes the max value from four corners (with 14×14×4 nodes), the dense layer (10 nodes, the output layer). The extracted input features are visualized in **Figure S15b**, where features from four corners (left up/down, right up/down) are max pooled as shown in **Figure S15c**. This is followed by the dense layer with output spikes shown in **Figure S15d**.

Spiking regulation, which inhibits a neuron in an inference propagation once it spikes, is applied to the feature layer to prevent features from low threshold neurons from spiking too long and temporally overlapping with features produced by high threshold neurons.

Lateral inhibition, which resets membrane potential of all neurons in the same layer if any of them spikes, is applied to the output layer neurons. This allows the output layer neurons produce spikes that reflect the instaneous features they receive from the convolutional layers, without being affected by the historical features.

Temporal separation of features in the feature layer allows independent feature to be correctly classified by a single object classifier. Early output spikes correspond to the object in the area

of lower threshold potential, or the area of interest, which receives attention thanks to spontenous threshold lowering. Latter output spikes correspond to the object in the area of higher threshold potential, or outside the area of interest.

Supporting Note 5. Analysis on internal state of STL neurons in the shallow feature layer

We convert image pixel values to spike intensity of repeat time spike (e.g. t_1, t_2, t_3) in the input layer. At each time step, these spikes propagate into a SCNN with pretrained weight. The SCNN feature layer neurons are implemented by YSZ:Ag memristors, whose thresholds reduce upon firing events. Before the spontaneous threshold lowering, these neurons have the same threshold as shown in **Figure S16a** left, where neurons within the area of interest and outside the area of interest take the same three time steps to accumulate the membrane potential to reach the threshold, as shown in **Figure S16a** right. Then the SCNN was fed with samples from the multi-object dataset, as shown in **Figure S17**, where two handwritten digits appear in different corners of the receptive field at different frequencies. After two epochs of inference, the neurons inside the area of interest experienced more spikes than those outside the area of interest, thus having lower thresholds as shown in **Figure S16b** left. Consequently, the neurons inside the area of interest take only two time steps to accumulate the membrane potential to fire (**Figure S16b** right upper), while the neurons outside the area of interest still take three time steps to fire (**Figure S16b** right lower). As such, the STL neurons in the feature layer practice the spatial attention according to the spatial locations of the multiple objects. The threshold of neurons in the area that objects appear more frequently (or so called area of interest) decreases, a manifestation of the spatial attention that the neurons within the area of interest spike faster than the rest. Such a spatial attention mechanism is physically achieved by the STL of YSZ:Ag memristor in a self-adaptive manner.

In addition to the self-adaptive spatial attention, the YSZ:Ag memristor-based STL neurons also reduce the latency in inference. Comparing neuron thresholds in **Figure S16b** and **S16c**, the average neuron thresholds decrease with epochs, leading to fewer time steps to spike for both neurons within and outside the area of interest and faster information processing of SCNN.

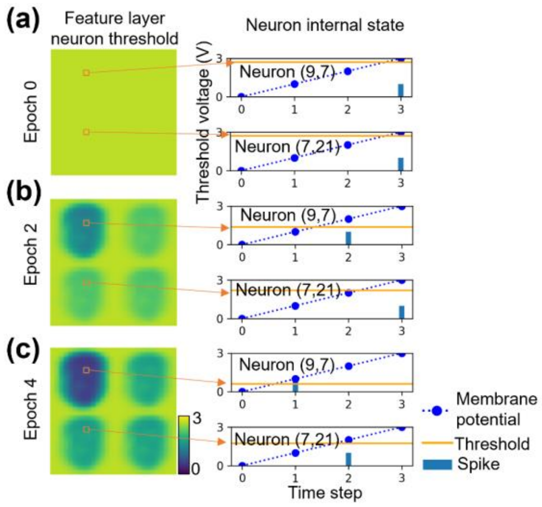

Figure S16. The threshold map of the STL neurons in the feature layer. The arrows highlight the internal state of neuron (9,7) that is within the area of interest and neuron (7,21) that is outside of the area of interest, at epoch 0 (a), 2 (b), 4 (c).

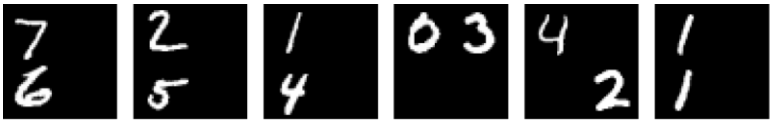

Figure S17. Large visual field dataset illustration, with two handwritten digits appearing at different locations. Here 6 examples are shown out of a total 60000 samples with 10000 randomly chosen test samples.

Supporting Note 6. Reliability and robustness of YSZ:Ag memristor for STLbased spatial attention.

1. Device-to-device threshold variation: First, we examined the impact of the device-to-device threshold variation on the STL-based spatial attention. We experimentally acquired the variation, as shown in **Figure S18a**, which was fitted to a Gaussian distribution with a mean 0.817V and a standard deviation 0.045V. In the simulation, the initial neural thresholds were sampled from the Gaussian distributions of the same mean and a varying standard deviation (from 0.740V to 0.940V). The multiple object classification accuracy as a function of the standard deviation is shown in **Figure S18b**. We find there is no clear degradation in the classification performance unless the standard deviation reaches 0.3V (for comparison, the experimental one is 0.045V). This indicates the initial neural thresholds variation imposes relatively less influence to the multi-object classification using SCNN with STL.

2. Device-to-device and cycle-to-cycle threshold lowering rate variation: We also tested the impact of device-to-device and cycle-to-cycle threshold lowering rate variation on STL-based spatial attention. **Figure S18c** shows the experimentally acquired distribution of threshold lowering rates, which was fitted to a Gaussian distribution with a mean 0.615 and a standard deviation of 0.129. The simulation samples the threshold lowering rates from a Gaussian distribution with the same mean and a varying standard deviation (from 0.444 to 0.816). According to the simulation result depicted in **Figure S18d**, the variation of the threshold lowering rate dose not degrade the accuracy of multiple-object classification of SCNN with STL significantly unless it goes beyond 0.2.

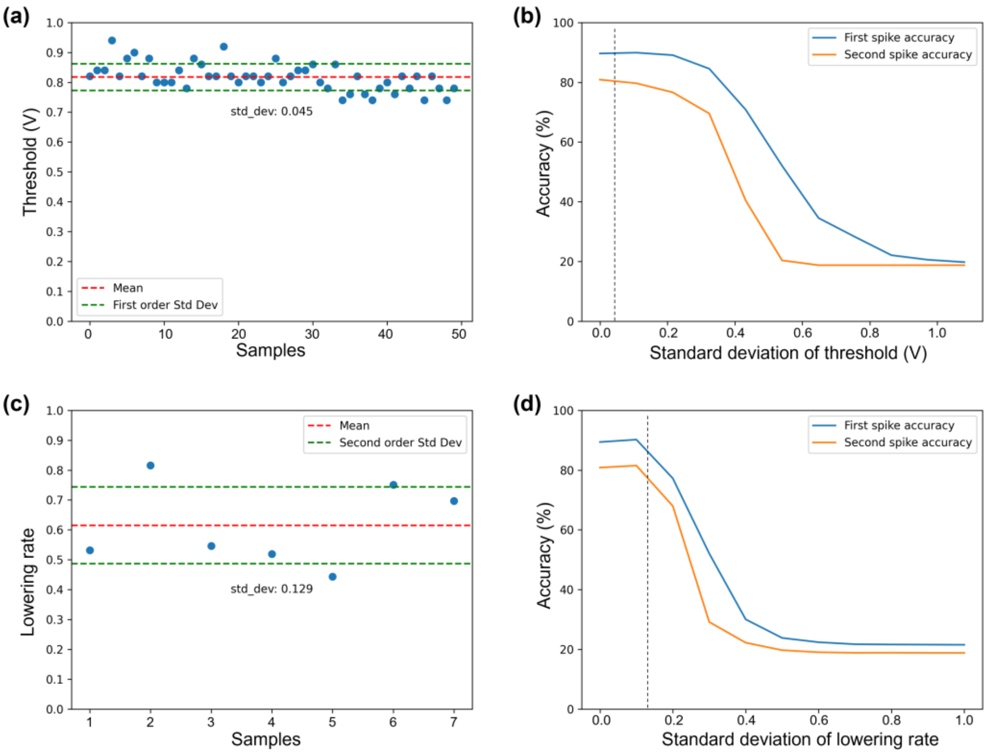

Figure S18. Impact of device variation on STL-based spatial attention. (a) Experimentally acquired device-to-device neural threshold distribution of YSZ:Ag memristors. (b) Multi-object classification accuracy as a function of the standard deviation of threshold distributions. (c) Experimentally acquired device-to-device and cycle-to-cycle threshold lowering rate distribution of YSZ:Ag memristors. (d) Multi-object classification accuracy as a function of the standard deviation of threshold lowering rate distributions.

3. The impact of choosing different time step: The choice of three-time step inference is to minimize the latency of SCNN interference with STL-based spatial attention. The initial time step is determined by the pulse width of input pulse and the initial threshold. With shorter input pulse, the SCNN requires more time steps to achieve the same learning performance under a same initial neural threshold. As shown in **Figure S19**, if the input pulse width is shortened by half, the membrane potential accumulates at the half speed and takes six-time step to spike. However, this does not affect the performance of the STL-based spatial attention, although incurring extra complexity in spike regulation. Thus, we choose three-time step for STL-based spatial attention.

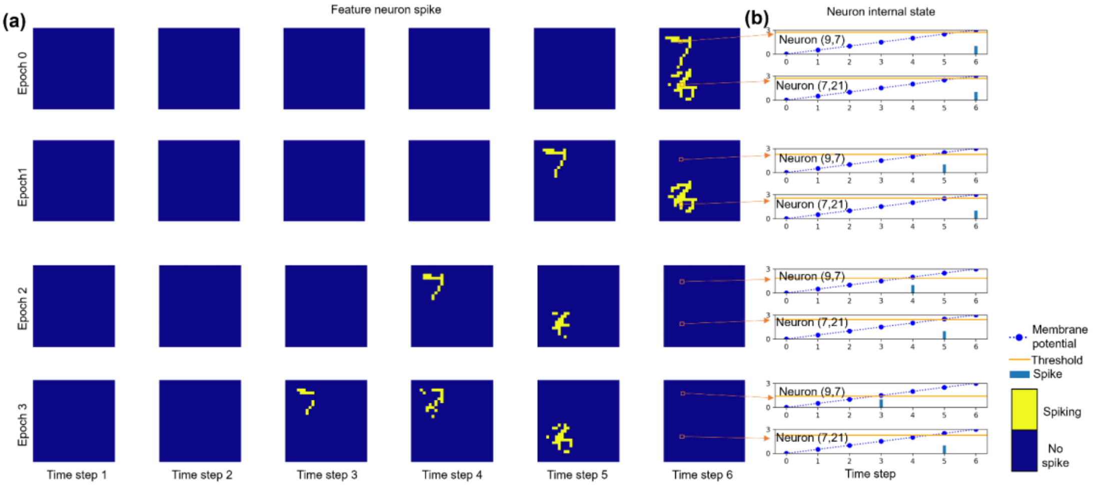

Figure S19. (a) Feature neuron spiking map with half pulse width and six-time steps. (b) Internal state of neuron (9,7) that is within the area of interest and neuron (7,21) that is outside of the area of interest.

Supporting Note 7. The ablation experiment without utilizing the STL neurons.

To evaluate the effectiveness of the STL neuron in implementing the spatial attention mechanism, we conducted an ablation experiment using neurons without STL. The results are presented in **Figure S20**, which shows the shallow feature layer with and without STL in **Figure S20a and S20b**, and the spikes of the classifier output with and without STL in **Figure S20c and S20d**. The resulting accuracy is illustrated in **Figure S20e**.

1. Performance boost: Figure S20e demonstrates the performance boost of SCNN achieved through the use of the STL neuron. The blue lines (solid and dashed) indicate the classification accuracy of the first classifier output spike corresponding to the object within the area of interest, while the orange lines (solid and dashed) indicate the classification accuracy of the second classifier output spike corresponding to the object outside the area of interest. (Initially, the accuracy of the second classifier output spike is lower than that of the first classifier output spike due to temporal overlap of spikes of the feature layer, as depicted in **Figure S20b** at Epoch 0.)

Both the first and second classifier output spikes exhibit higher accuracy in the solid lines (with STL neuron) than in the dashed lines (without STL neuron), clearly indicating the effectiveness of the STL neuron. This can be explained by examining **Figure S20a-S20d**: the SCNN without the STL neuron had a fixed threshold in the feature layer, as shown in **Figure S20a**, causing the spikes of the feature layer of different objects to appear at the same time. As a result, the simple classifier was unable to recognize both objects (within and outside the area of interest) during the inference, as depicted in **Figure S20c**. Conversely, the SCNN with the STL neuron can adaptively lower the neuron threshold, as shown in **Figure S20b**. During inference, the neuron threshold in the area of interest decreases more because the neurons

spike more frequently than those outside the area of interest. Consequently, the feature of the object inside the area of interest spikes earlier, temporally separating it from the feature of the object outside the area of interest. This leads to the classifier spiking at different time steps, as shown in **Figure S20d**. For example, at Epoch 2, the classifier outputs the first spike at t_2, corresponding to the object inside the area of interest (Digital 7, in the illustrated case). At t_3, the classifier outputs two spikes due to the presence of the object outside the area of interest, and one is arbitrarily chosen to evaluate the classification accuracy of the second object. In summary, it's observed that both the objects within and outside the area of interest experience recognition accuracy improvement compared to the case without STL neurons, thereby solving the feature "binding problem" in a self-adaptive manner.

2. The response time reduction: Figure S20a-S20d demonstrates that the SCNN with STL neurons generates spikes in the feature layer and classifier at earlier time steps compared to the SCNN without STL neurons. For instance, at Epoch 8, the SCNN with STL neurons generates spikes in the feature layer and classifier at t_1 (t_2) for the object within (outside) the area of interest (**Figure S20b and S20d)**), whereas the SCNN without STL generates spikes for the feature layer and classifier at t_3 (**Figure S20a and S20c**). This is because a lower threshold of STL neuron enables a quicker response of the feature layer neurons, leading to a reduction in SCNN latency.

In summary, the SCNN without STL neurons behaves similarly to the pristine state of the SCNN with STL, where all the neuron thresholds are still the same.

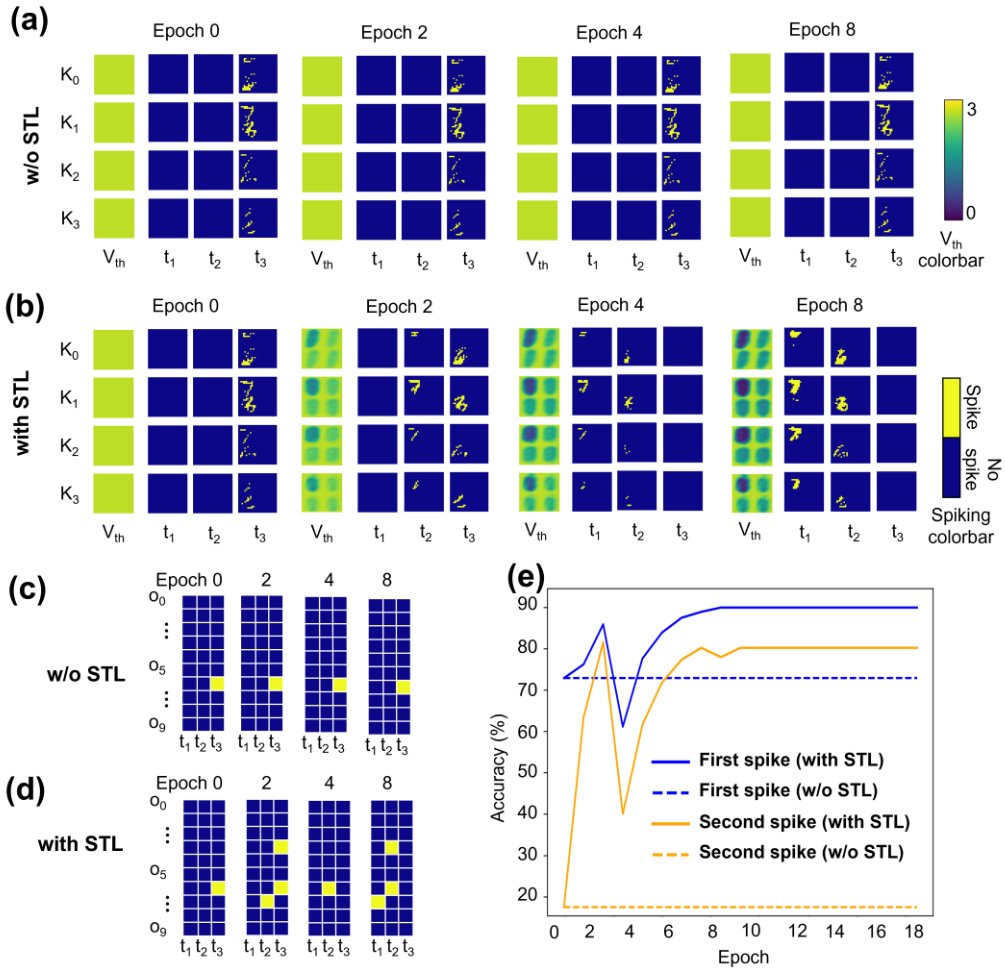

Figure S20. Ablation experiment on SCNN with and without STL neurons. (a) and (b) Threshold potential and spike maps of the shallow feature layer neurons without (a) / with (b) STL neurons. (c) and (d) Spike maps of output layer neurons without (c) / with (d) STL neuron. (e) The classification accuracy of the first classifier output spike (for the object within the area of interest) and the second classifier output spike (for the object outside the area of interest) without (dashed line) / with (solid line) STL neurons.

References

[1] J. H. Yoon, Z. Wang, K. M. Kim, H. Wu, V. Ravichandran, Q. Xia, C. S. Hwang, J. J. Yang, *Nat. Commun.* **2018**, 9, 417.

[2] X. Wang, P. Huang, Z. Dong, Z. Zhou, Y. Jiang, R. Han, L. Liu, X. Liu, J. Kang, presented at 2018 International Symposium on VLSI Technology, Systems and Application (VLSI-TSA) **2018**.

[3] A. Shaban, S. S. Bezugam, M. Suri, *Nat. Commun.* **2021**, 12, 4234.

[4] I. Muńoz-Martín, S. Bianchi, S. Hashemkhani, G. Pedretti, D. Ielmini, presented at 2020 IEEE International Symposium on Circuits and Systems (ISCAS) **2020**.

[5] Y. Xu, S. Gao, Z. Li, R. Yang, X. Miao, *Adv. Intell. Syst.* **2022**, 2200210.

[6] Z. Xie, X. Zhu, W. Wang, Z. Guo, Y. Zhang, H. Liu, C. Sun, M. Tang, S. Gao, R. W. Li, *Adv. Electron. Mater.* **2022**, 8, 2200334.

[7] Y. Yang, P. Gao, L. Li, X. Pan, S. Tappertzhofen, S. Choi, R. Waser, I. Valov, W. D. Lu, *Nat. Commun.* **2014**, 5, 4232.

[8] X. Tian, S. Yang, M. Zeng, L. Wang, J. Wei, Z. Xu, W. Wang, X. Bai, *Adv. Mater.* **2014**, 26, 3649.

[9] Y. Yang, P. Gao, S. Gaba, T. Chang, X. Pan, W. Lu, *Nat. Commun.* **2012**, 3, 732.

[10] W. Wang, M. Wang, E. Ambrosi, A. Bricalli, M. Laudato, Z. Sun, X. Chen, D. Ielmini, *Nat. Commun.* **2019**, 10, 81.

[11] W. Wang, E. Covi, Y.-H. Lin, E. Ambrosi, A. Milozzi, C. Sbandati, M. Farronato, D. Ielmini, *IEEE Trans. Electron Devices* **2021**, 68, 4342.

[12] W. Wang, M. Laudato, E. Ambrosi, A. Bricalli, E. Covi, Y.-H. Lin, D. Ielmini, *IEEE Trans. Electron Devices* **2019**, 66, 3795.

[13] S. Kobayashi, in *Encyclopedia of Neuroscience*, DOI: https://doi.org/10.1016/B978-008045046-9.01559-X (Ed: L. R. Squire), Academic Press, Oxford **2009**, p. 325.

[14] K. Tanaka, *Annu. Rev. Neurosci.* **1996**, 19, 109.

[15] A. Treisman, *Curr. Opin. Neurobiol.* **1996**, 6, 171.

[16] R. VanRullen, S. J. Thorpe, *Neurocomputing* **1999**, 26, 911.

[17] J. K. Eshraghian, M. Ward, E. Neftci, X. Wang, G. Lenz, G. Dwivedi, M. Bennamoun, D. S. Jeong, W. D. Lu, arXiv preprint arXiv:2109.12894 **2021**.

[18] Y. LeCun, L. Bottou, Y. Bengio, P. Haffner, Proc. IEEE **1998**, 86, 2278.

[19] L. F. Abbott, *Brain Res. Bull.* **1999**, 50, 303.

[20] E. M. Izhikevich, *IEEE Trans. on Neural Networks* **2003**, 14, 1569.

[21] A. L. Hodgkin, A. F. Huxley, *J. Physiol.* **1952**, 117, 500.
